# Electrically Assisted Thermal Stamping of Tunable Carbon‐Based Nanofilms for Direct Fabrication of Hydrophobic, Energy Harvesting, and Sensing Devices

**DOI:** 10.1002/adma.202516478

**Published:** 2026-01-08

**Authors:** Byungseok Seo, Yong Choi, Gajendra S. Shekhawat, Minjoong Shin, Kunmo Koo, Xiaobing Hu, Jiheon Kim, Wonjoon Choi, Xinqi Chen, Vinayak P. Dravid

**Affiliations:** ^1^ The NUANCE Center Northwestern University Evanston Illinois USA; ^2^ Department of Materials Science and Engineering Northwestern University Evanston Illinois USA; ^3^ School of Mechanical Engineering Korea University Seoul Republic of Korea; ^4^ Department of Chemical and Biological Engineering Northwestern University Evanston Illinois USA; ^5^ Department of Mechanical Engineering Northwestern University Evanston Illinois USA; ^6^ International Institute for Nanotechnology Northwestern University Evanston Illinois USA

**Keywords:** humidity sensing, hydrophobic coating, lithography‐free fabrication, nanofilm, triboelectric nanogenerator

## Abstract

The demand for multifunctional thin‐film devices has accelerated the development of scalable fabrication techniques capable of precisely controlling composition, structure, and patterning. However, conventional approaches rely on complex, multi‐step processing and harsh conditions, limiting their applicability. Herein, we report a single‐step, electrically assisted thermal stamping (EATS) method for the direct and scalable fabrication of carbon‐based nanofilms, including reduced graphene oxide (rGO), polytetrafluoroethylene (PTFE), and their nanocomposite films. By applying localized Joule heating to PTFE‐embedded carbon paper under ambient conditions, EATS induces graphite exfoliation, GO reduction, and PTFE incorporation, thereby eliminating the need for multi‐step processing or harsh environments. The resulting films exhibit tunable thickness, morphology, and composition, governed by EATS power density and duration. EATS enables spatially selective patterning without the need for complex lithographic techniques. The versatility of EATS is demonstrated through the direct fabrication of functional devices: (i) hydrophobic coatings with contact angles tunable from 44.3° to 109.8°, (ii) triboelectric nanogenerators achieving 10.11 mW cm^−3^ power density with <2.8% variation over 10 000 cycles, and (iii) humidity sensors exhibiting a 3.56% sensing error across 50–100% relative humidity. These results establish EATS as a powerful, lithography‐free fabrication platform for multifunctional thin‐film devices, offering a generalizable strategy for next‐generation electronics.

## Graphical abstract

Schematic illustration of the electrically assisted thermal stamping (EATS) method for direct fabrication of carbon‐based nanofilms and their multifunctional applications. Localized Joule heating triggers simultaneous exfoliation, reduction, and fluoropolymer incorporation under ambient conditions, yielding tunable carbon‐based thin‐film coatings, including rGO, PTFE, and rGO/PTFE. This single‐step, rapid, and lithography‐free process enables direct integration into diverse thin‐film devices, including hydrophobic surface engineering, energy harvesters, and sensors, highlighting the scalability and versatility of EATS for next‐generation electronics and environmental interfaces.

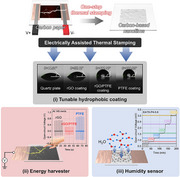

## 1 Introduction

Film‐type devices play a critical role in modern technologies due to their lightweight form factor, large‐area compatibility, and adaptability to diverse substrates [1, 2, 3, 4]. They have been widely utilized in electronics, thermal management, surface coatings, energy conversion, and sensing applications, where device performance is strongly influenced by film thickness, surface morphology, and compositional uniformity [5, 6, 7, 8, 9]. In particular, integrating multiple functional layers within a single film structure allows for the realization of compact and multifunctional systems with tailored electrical, thermal, and chemical properties [10, 11, 12]. However, achieving precise control over these features typically demands multi‐step fabrication workflows and complex processing environments, which limits both scalability and reproducibility [13, 14, 15]. Thus, there is a growing demand for efficient fabrication strategies that provide tunable material properties with minimal processing complexity to meet the requirements of next‐generation device technologies [16, 17].

Conventional fabrication techniques for film‐based devices, such as chemical vapor deposition, spin coating, inkjet printing, solution casting, and electrothermal processing, have been employed to engineer functional thin films with controlled material properties [18, 19, 20, 21, 22, 23]. While these methods provide precise control over individual components, they often require high temperatures, vacuum systems, or aggressive chemicals, particularly when multiple components are incorporated [24, 25, 26]. Such segmented approaches increase production complexity and frequently result in material distribution inconsistencies, which compromises the structural cohesion and functional uniformity across large‐area films [27, 28]. Furthermore, integrating multiple functional layers with differing thermal, chemical, or mechanical properties often necessitates extensive post‐processing treatments, which can inadvertently induce interfacial defects, material degradation, or phase separation [29, 30, 31, 32]. These challenges are further exacerbated in composite systems where co‐processing of chemically incompatible constituents is required [33, 34, 35, 36].

Among candidate materials for multifunctional film‐type devices, carbon‐based materials, such as reduced graphene oxide (rGO) and polytetrafluoroethylene (PTFE), have attracted considerable interest due to their complementary properties and broad functional applicability [37, 38, 39]. rGO offers high electrical conductivity, thermal stability, and mechanical robustness, making it ideal for energy storage, sensors, and flexible electronics [40, 41, 42]. In contrast, PTFE is renowned for its excellent hydrophobicity, dielectric strength, and chemical resistance, enabling its use in energy harvesting, surface modification, or protective coatings for electronic systems [43, 44, 45]. Their combination in rGO/PTFE hybrid films provides a synergistic integration of electrical, mechanical, and surface functionalities, which is particularly promising for advanced devices such as self‐cleaning nanogenerators, multifunctional coatings, and environmental sensors [46, 47, 48, 49, 50].

However, fabricating high‐quality rGO or PTFE films individually remains nontrivial, as each material demands distinct and often stringent processing conditions, such as long‐duration high‐temperature thermal reduction or chemical treatments lasting several hours for rGO, and vacuum environments, solvent dispersion, or sintering for high‐molecular‐weight PTFE, that are typically incompatible with simple, scalable, or ambient‐condition manufacturing [51, 52]. Co‐processing rGO and PTFE into hybrid films is even more challenging due to their fundamental differences in surface chemistry and process compatibility, often resulting in poor dispersion, weak interfacial adhesion, and phase segregation [53]. Despite their complementary properties and potential for multifunctional applications, current fabrication techniques struggle to produce either uniform single‐component films or well‐integrated rGO/PTFE composites with precise thickness control, spatial uniformity, and compositional tunability [54]. Conventional approaches using spin coating and solution casting typically require sequential deposition steps to integrate multiple components, which can lead to inhomogeneity, interface defects, and material loss, ultimately degrading device performance [55, 56, 57]. Furthermore, pre‐ and post‐treatment steps intended to promote interfacial bonding between rGO and PTFE often involve prolonged thermal or chemical exposure that can degrade the structural and functional integrity of the composite, especially for thermally or chemically sensitive constituents [58, 59]. Therefore, a simplified, single‐step method capable of selectively or simultaneously producing rGO, PTFE, and rGO/PTFE films in a controlled manner would be highly advantageous for the scalable fabrication of functional thin‐films, improving not only material uniformity and integration but also enabling diverse applications across energy, sensing, and protective interfaces.

**FIGURE 1:**
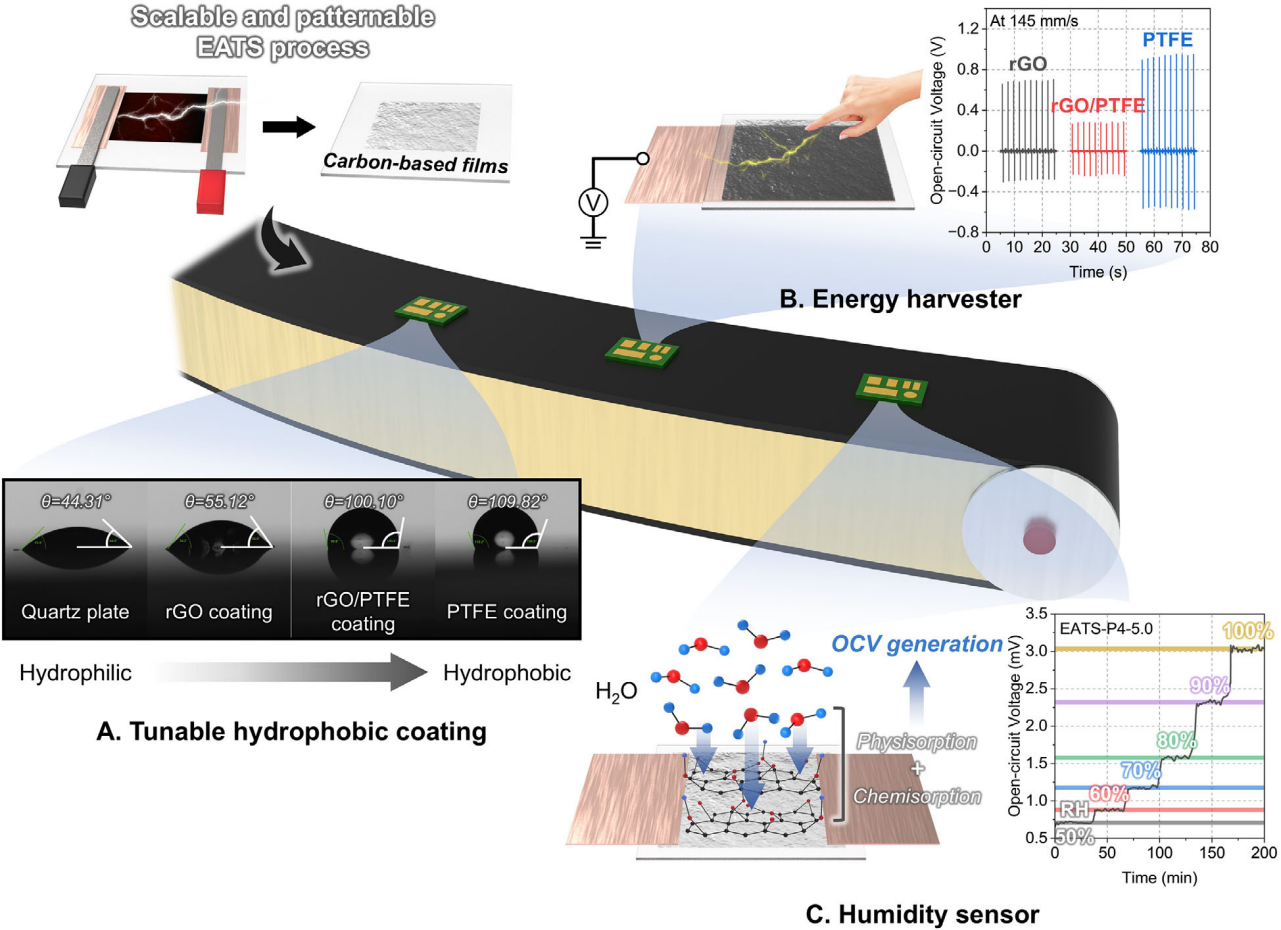
Schematic illustration of the one‐step electrically assisted thermal stamping (EATS) process for scalable and patternable fabrication of carbon‐based nanofilms. The EATS method enables direct integration of the films into multifunctional devices, including (A) tunable hydrophobic coatings with contact angle modulation, (B) energy harvesters based on mechano‐electric conversion, and (C) humidity sensors detecting water‐induced open‐circuit voltage (OCV), all without the need for bulky equipment or complex processing environments.

Here, we introduce a novel single‐step fabrication strategy, electrically assisted thermal stamping (EATS), for the direct synthesis of carbon‐based nanofilms under ambient conditions within a few seconds (Figure 1). By applying localized Joule heating to carbon paper (CP) embedded with PTFE, EATS simultaneously induces graphite exfoliation, rGO formation, and PTFE incorporation without the need for vacuum environments, long‐duration thermal treatment, or multiple processing. The process allows precise control over film thickness, sp^2^/sp^3^ carbon ratio, and PTFE content through simple modulation of power density and duration, while also enabling direct spatial patterning without complex lithographic steps. To demonstrate its practical applicability, EATS‐fabricated films were directly integrated into various functional devices, including tunable hydrophobic surface coatings, mechano‐electric nanogenerators, and ambient humidity sensors. Beyond carbon‐based materials, the EATS platform is also potentially applicable to other energy‐relevant 2D layered materials, such as MoS_2_, WS_2_, and BN, which possess a stacked lamellar structure similar to graphite and can be exfoliated under thermal stimulation, thus offering a versatile route for fabricating diverse functional nanofilms for surface engineering, energy, and sensing applications. These demonstrations establish EATS as a scalable, tunable, and lithography‐free fabrication platform for next‐generation multifunctional thin‐film devices, offering a compelling alternative to conventional multi‐step manufacturing routes.

## 2 Results and Discussion

### 2.1 EATS for the Fabrication of Tunable Carbon‐Based Nanofilms

**FIGURE 2:**
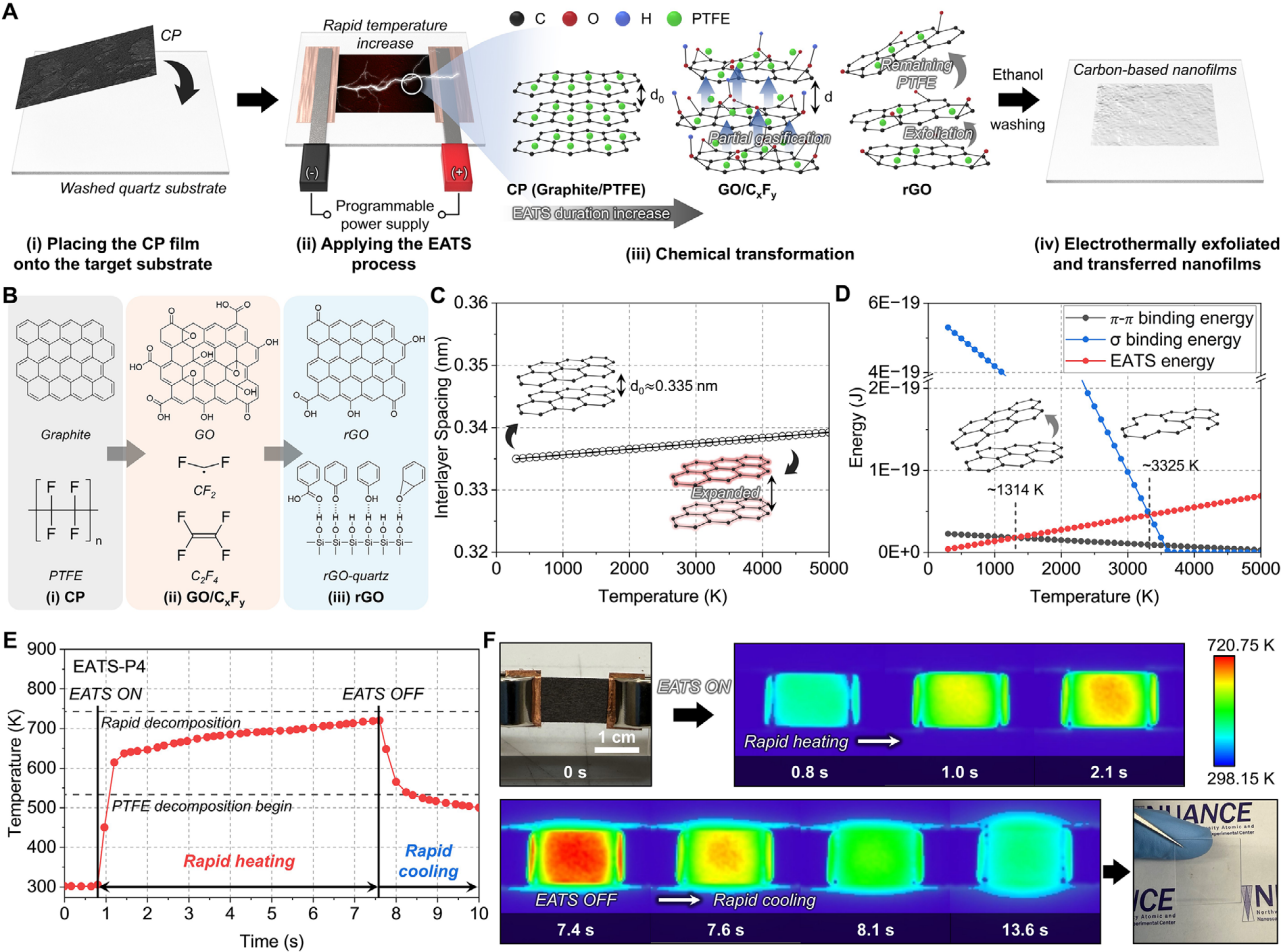
Working mechanism of carbon‐based nanofilm fabrication via the EATS process. (A) Schematic illustration of the fabrication steps: (i) placement of the CP film onto a pre‐cleaned quartz substrate, (ii) application of the EATS process via localized Joule heating, (iii) thermally driven chemical transformations including graphite exfoliation and PTFE decomposition, and (iv) formation and transfer of carbon‐based films onto the substrate following ethanol rinsing. (B) Molecular structures of (i) the initial CP composed of graphite and PTFE, (ii) intermediate GO/C_x_F_y_ species formed during the EATS process, and (iii) the resulting rGO structure integrated with the quartz surface. (C) Theoretically calculated expansion of graphite interlayer spacing as a function of temperature induced by EATS. (D) Comparison of energy delivered by EATS with the *π–π* and σ bond energies of graphite over a range of temperatures, revealing an optimal temperature window (∼1314–3325 K) where *π–π* bonds are disrupted while preserving σ bonds intact, enabling efficient exfoliation without structural collapse. (E) Temporal temperature profile of CP under EATS‐P4 condition for 6.8 s, illustrating rapid heating, onset of PTFE decomposition, and subsequent rapid cooling. (F) Corresponding infrared thermal images showing uniform and localized heat distribution across the CP surface during the EATS process.

To fabricate carbon‐based nanofilms, an EATS method was developed based on Joule heating, which directly converts electrical energy into thermal energy (Figure 2A). This process rapidly elevates the system temperature, triggering interlayer exfoliation of graphite and subsequent deposition of exfoliated species onto target substrates. Starting with commercially available carbon paper (CP) consisting of graphite that is uniformly encapsulated by polytetrafluoroethylene (PTFE), nanocomposite films were successfully synthesized within a few seconds under ambient conditions. As shown in Figure 2A (i), a CP strip (2 cm × 1 cm) was placed onto a quartz substrate pre‐cleaned with acetone and deionized (DI) water. Copper tapes were affixed to both ends of the CP with a spacing of 1.5 cm, and electrical alligator clips connected the system to a programmable power supply (Figure 2A (ii)). Joule heating during the EATS process caused a rapid temperature rise, resulting in significant expansion of the graphite interlayer spacing (d) (Figure 2A (iii)). Once the local temperature exceeded the PTFE decomposition threshold, partial gasification of PTFE generated volatile fluorocarbon species that temporarily increased the local pressure within confined graphite–PTFE contact regions near the substrate. Despite the high porosity of the carbon paper, this rapid heating and partial interfacial sealing created transient overpressure before the gases could escape through the macropores, mechanically driving interlayer exfoliation. Because the EATS process occurs within a few seconds, a portion of PTFE that does not fully decompose remains as residual PTFE surrounding the exfoliated graphite regions. This synergistic combination of transient thermal expansion and partial gas evolution enabled efficient exfoliation. Simultaneously, high temperatures triggered partial oxidation of graphite into a graphene oxide (GO) intermediate, followed by in situ reduction to reduced graphene oxide (rGO). The oxidation step is facilitated by thermally activated oxygen species originating from ambient air confined within the porous carbon paper network, which can transiently react with exposed graphite edges and defect sites during rapid Joule heating [60, 61]. Exfoliated species were subsequently deposited onto the quartz surface through van der Waals (vdW) and hydrogen bonding interactions. After rinsing with ethanol, the final carbon films were obtained (Figure 2A (iv)).

As illustrated in Figure 2B, pristine CP consists of stacked graphite with uniformly embedded PTFE particles. Under thermal stress, PTFE decomposes via homolytic cleavage of C─C bonds to generate difluorocarbene (CF_2_) radicals (Figure 2B (i)–(ii)):

(1) $${\left(C_{2}F_{4}\right)}_{n}\to \mathrm{nC}F_{2}$$

These highly reactive CF_2_ radicals dimerize to form tetrafluoroethylene (C_2_F_4_) gas (Figure 2B (ii)):

(2) $$2CF_{2}\to C_{2}F_{4}$$

The volatile C_2_F_4_ molecules diffuse between graphite layers, weakening vdW interactions and promoting interlayer delamination. In parallel, the elevated temperatures partially oxidize graphite into GO through reactions with ambient oxygen and moisture, introducing oxygen‐containing functional groups such as epoxides, hydroxyls, and carboxyls onto the graphitic lattice. Subsequently, CF_2_ radicals act as in situ reductants, chemically reacting with these oxygenated moieties and restoring the sp^2^‐hybridized carbon frameworks (Figure 2B (iii)). This integrated mechanism enables single‐step fabrication of carbon‐based films under ambient conditions with tunable composition and structure.

To validate the exfoliation and transfer mechanism, theoretical analyses were performed using molecular‐scale calculations (Figure 2C,D). The temperature‐dependent interlayer binding energy per particle (E_π‐π_) and interlayer spacing (d) were modeled as follows:

(3) $$E_{\pi -\pi }=4\varepsilon \left[{\left\{\frac{\sigma }{d\left(T\right)}\right\}}^{12}-{\left\{\frac{\sigma }{d\left(T\right)}\right\}}^{6}\right]$$

(4) $$d\;\left(T\right)=d_{0}\;\left\{1+\alpha \left(T-T_{0}\right)\right\}$$

Here, ε is the potential well depth, σ is the effective atomic diameter, T_0_ is the room temperature, d_0_ is the interlayer spacing at T_0_ (0.335 nm), α is the thermal expansion coefficient, and T is the system temperature [62, 63]. As temperature increased to 4998 K, the interlayer spacing expanded to 0.339 nm, while the interlayer binding energy decreased from 2.28 × 10^−20^ J to 3.03 × 10^−21^ J, indicating substantial weakening of interlayer cohesion (Figure 2C,D) [64]. Additionally, the thermal energy per particle generated during the EATS process (E_EATS_) and the σ bond (C─C) dissociation energy (E_σ_) were estimated as follows:

(5) $$E_{\mathrm{EATS}}=k_{B}\;T$$

(6) $$E_{\sigma }\;\left(T\right)=E_{\sigma ,\;0}\;-\beta T$$

where k_B_ is the Boltzmann constant, E_σ,0_ is the σ bond dissociation energy at room temperature, and β is the temperature coefficient [65, 66, 67]. As temperature rose to 4998 K, the thermal energy increased from 4.11 × 10^−21^ J to 6.90 × 10^−20^ J, while the σ bond energy dropped from 5.30 × 10^−19^ J to nearly zero around 3698 K, suggesting significant destabilization of covalent bonds at extreme heat. From this analysis, a critical threshold temperature of ∼1314 K was identified above which interlayer exfoliation becomes thermodynamically favorable, yet the system remains structurally stable up to ∼3325 K, below the σ bond breaking limit. This analysis supports the feasibility of high‐temperature exfoliation without catastrophic lattice degradation, supporting the thermal robustness of the EATS process.

To enable tunable film fabrication, EATS power density was varied from 70 to 140 W/cm^2^, defined across ten levels: P1 (70.0 W/cm^2^), P2 (73.3 W/cm^2^), P3 (76.7 W/cm^2^), P4 (80.0 W/cm^2^), P5 (83.3 W/cm^2^), P6 (86.7 W/cm^2^), P7 (100 W/cm^2^), P8 (113 W/cm^2^), P9 (127 W/cm^2^), and P10 (140 W/cm^2^). The process duration was controlled within a range of 1.0 to 7.0 s. Each film is denoted as EATS‐power density‐duration, where the power density is represented by P1–P10, and the duration is specified by the set time in seconds. For example, EATS‐P4‐5.0 represents a film processed at 80.0 W/cm^2^ for 5.0 s.

The surface temperature of CP under various EATS conditions was monitored to characterize the thermal response (Figure 2E; Figure S1). Although the buried CP and substrate interface cannot be directly visualized during the rapid Joule‐heating event, the combination of surface temperature measurements, threshold‐energy modeling, and PTFE decomposition analysis provides a coherent set of thermal and mechanistic evidence that collectively enables a reliable reconstruction of the interfacial environment. Under EATS‐P4, temperature rapidly rose from ambient to 533 K within 0.28 s, reaching the PTFE decomposition onset (Figure 2E) [68]. This rapid heating capability enables uniform decomposition and volatilization of PTFE embedded within the graphite interlayers, generating internal pressure that facilitates layer exfoliation. Notably, this mechanism indicates that effective exfoliation can be achieved even below the theoretical threshold (∼1314 K) (Figure 2D; Note S1). Consequently, the system operates at comparatively moderate temperatures, minimizing thermal stress while preserving graphitic integrity and film uniformity, thereby enhancing process stability and reproducibility. After 6.80 s, the peak temperature reached 721 K, followed by rapid cooling upon power termination. Importantly, infrared imaging confirmed uniform thermal distribution across the entire CP surface, which helps prevent localized overheating and ensures consistent thermal expansion and exfoliation (Figure 2F). This uniform heating is particularly advantageous for homogenous film formation across the substrate.

Comparatively, under EATS‐P1, the PTFE decomposition temperature (673.15 K) was not reached until 5.80 s, with a peak temperature of only 680 K for 6.80 s (Figure S2A,B). Conversely, under EATS‐P7, the decomposition threshold was surpassed within just 0.44 s, with a peak temperature of 846 K (Figure S2C,D). In addition, thermogravimetric analysis–mass spectrometry (TGA–MS) of the PTFE‐containing carbon paper revealed a pronounced rapid‐decomposition peak near ∼743 K, confirming active PTFE pyrolysis under conditions directly comparable to those reached during EATS (Figure S3). Consistent with this behavior, TGA measurements performed under purified air conditions showed that PTFE‐containing CP undergoes substantially greater mass loss than PTFE‐free CP at elevated temperatures, indicating dominant PTFE volatilization accompanied by oxidation and etching of the carbon framework (Figure S4). Combined with prior literature reporting that PTFE begins to dissociate at temperatures as low as 533 K, the TGA–MS results reinforce that the volatilization and gas evolution observed in EATS arise from genuine thermal decomposition, thereby supporting the proposed exfoliation and nanofilm‐formation mechanism. These distinct thermal response profiles highlight the highly tunable thermal behavior of EATS, where film characteristics can be precisely controlled by modulating power density and duration. In particular, when combined with threshold‐energy modeling, real‐time surface thermography, and TGA–MS–verified PTFE decomposition windows, these thermal profiles provide indirect yet comprehensive evidence of the interfacial heating and gas‐assisted exfoliation processes that would otherwise be probed through direct cross‐sectional observation. Together, these datasets coherently validate that rapid Joule heating, PTFE volatilization, and thermally driven expansion act synergistically to drive nanofilm formation during EATS. This tunability enables customized synthesis of graphitic films with diverse structural and compositional characteristics, offering a robust platform for application‐specific material design.

### 2.2 Physicochemical Characterization of Carbon‐Based Films for Tunable EATS Platforms

**FIGURE 3:**
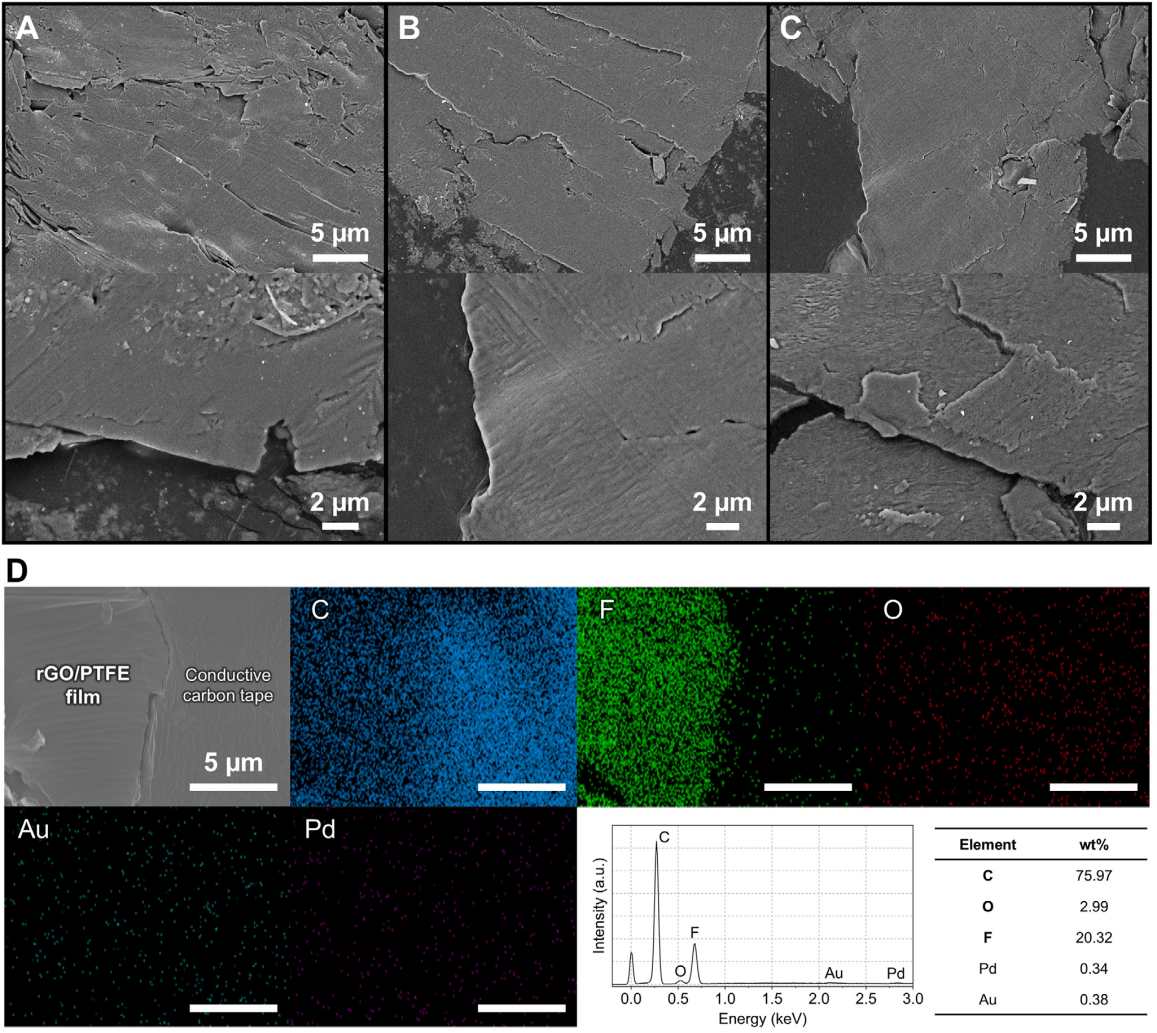
SEM and EDS analyses of carbon‐based nanofilms. SEM images of (A) EATS‐P4‐5.0, (B) EATS‐P4‐7.0, and (C) EATS‐P7‐3.0 films, representing rGO, rGO/PTFE, and PTFE surfaces, respectively, for surface morphology analysis. (D) EDS mapping and elemental composition analysis of the rGO/PTFE film (EATS‐P4‐7.0), showing the spatial distribution of carbon (C), fluorine (F), oxygen (O), gold (Au), and palladium (Pd). The corresponding spectrum and elemental weight percentages are presented in the graph and table, confirming the co‐existence of rGO and PTFE within the composite structure.

The physicochemical properties of carbon‐based nanoflms synthesized via the EATS process were systematically characterized using scanning electron microscopy (SEM), energy‐dispersive X‐ray spectroscopy (EDS), Raman spectroscopy, and X‐ray photoelectron spectroscopy (XPS). Figure 3 presents the surface morphologies of films prepared under various EATS conditions. For SEM analysis, films were mechanically delaminated from quartz substrates using a razor blade and transferred onto conductive carbon tape. Prior to EATS processing, the pristine CP exhibited a porous carbon fiber network embedded with PTFE, which served both as a conductive scaffold for Joule heating and a dual precursor, providing fluorinated species from PTFE and graphitic carbon from graphite (Figure S1). The EATS‐P4‐5.0 sample, consisting primarily of rGO, exhibited a smooth and continuous surface with low roughness, suggesting uniform deposition of exfoliated graphene layers under moderate thermal input (Figure 3A). In the EATS‐P4‐7.0 sample, incorporation of PTFE produced a denser and more compact morphology, where PTFE filled interlayer voids and contributed to improved structural cohesion, while maintaining overall surface uniformity (Figure 3B). In contrast, the EATS‐P7‐3.0 film, dominated by PTFE, displayed a smooth yet featureless surface with minimal graphitic texture, reflecting PTFE volatilization and redeposition into a continuous fluoropolymer film under high power input (Figure 3C).

Elemental distribution within the rGO/PTFE composite synthesized under EATS‐P4‐7.0 conditions was examined via EDS mapping (Figure 3D). Carbon and fluorine were prominently distributed across the film, confirming the successful integration of rGO and PTFE. The low oxygen content indicates efficient reduction of oxygen‐containing functional groups, indicating the conversion of GO to rGO. Trace amounts of gold and palladium originated from the sputter‐coating for EDS mapping. Quantitative EDS analysis revealed 75.97 wt.% carbon, 20.32 wt.% fluorine, and 2.99 wt.% oxygen. Additionally, region‐of‐interest (ROI)–based intensity analysis of the EDS maps yielded an F/C ratio of ∼1.63 for the rGO/PTFE film, which is significantly higher than that of the underlying carbon tape (∼0.0335), further confirming localized PTFE enrichment in the composite region (Figure S5). It is noted that this mapping‐based ratio is intended for relative comparison rather than absolute stoichiometric quantification. The substantial fluorine content further verifies PTFE incorporation, while the low oxygen content reinforces successful rGO reduction. The coexistence of rGO and PTFE implies the presence of both sp^2^‐hybridized carbon (C═C) from rGO and sp^3^‐hybridized carbon (C─C) from PTFE, resulting in a hybrid composite structure. These results highlight the tunability of the EATS process, which allows modulation of films ranging from predominantly sp^2^ carbon (rGO) to sp^3^‐rich PTFE structures with distinct structural and surface characteristics.

**FIGURE 4:**
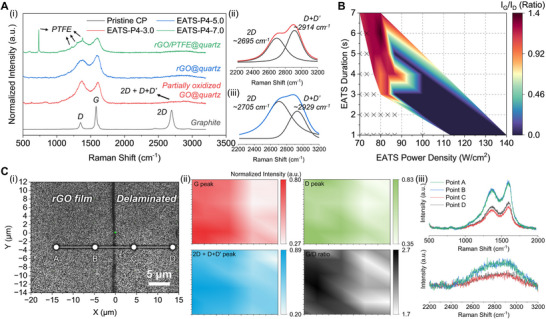
Raman spectroscopy analysis of carbon‐based nanofilms. (A) Raman spectra of pristine CP, EATS‐P4‐3.0, EATS‐P4‐5.0, and EATS‐P4‐7.0, corresponding to graphite, partially oxidized GO, rGO, and rGO/PTFE, respectively. (i) Comparative raw spectra. Peak deconvolution of the 2D and D+D′ bands using Voigt functions for (ii) EATS‐P3‐3.0 and (iii) EATS‐P4‐5.0, respectively. (B) Contour map of G/D peak intensity ratio across various EATS power densities and durations. ‘X’ markers denote conditions under which carbon film formation cannot be achieved. (C) Raman mapping of the rGO film fabricated at EATS‐P4‐5.0 condition. (i) Optical image highlighting the boundary between intact film and delaminated region, with points A–D marked for spectral sampling. (ii) Spatially resolved intensity maps of the G band, D band, 2D+D+D′ band, and corresponding G/D ratio. (iii) Representative Raman spectra from points A–D in (i).

Raman spectroscopy provided further insight into carbon hybridization states (sp^2^ vs. sp^3^) and fluorine‐related vibrational modes associated with PTFE (Figure 4). Raman spectra were obtained for pristine CP and carbon films fabricated under EATS‐P4‐3.0, EATS‐P4‐5.0, and EATS‐P4‐7.0 conditions on quartz substrates (Figure 4A (i)). The pristine CP exhibited a sharp G band (∼1580 cm^−1^) and a broad 2D band (∼2695 cm^−1^), characteristic of graphitic structures with low defect density and highly ordered sp^2^ carbon domains [69, 70]. Under EATS‐P4‐3.0, initial exfoliation and chemical oxidation of graphite led to the formation of partially oxided GO, as evidenced by the increased D band (∼1367 cm^−1^) intensity and a G/D ratio of 0.94. This indicates partially oxygenated defects and disrupted sp^2^ carbon networks. The emergence of a 2D+D+D' combination band (2600–3000 cm^−1^) further supports structural disorder and functional group incorporation [71]. The relatively weak intensity of the 2D+D+D' band compared with the D and G bands arises from the layer stacking and structural disorder of thermally exfoliated rGO sheets, which suppress the double‐resonance scattering processes responsible for these overtones [71, 72, 73]. At EATS‐P4‐5.0, the G/D ratio increased to 1.24, signifying effective reduction of GO to rGO. This is attributed to CF_2_ radicals reacting with oxygen‐containing functional groups, restoring the sp^2^ carbon framwork. Under EATS‐P4‐7.0, in addition to prominent rGO features, distinct PTFE‐related peaks were observed at 731, 1215, 1300, and 1380 cm^−1^, indicating an incorporation of PTFE (Figure 4A (i)) [74]. The coexistence of rGO and PTFE signals confirms the formation of a composite structure, where residual PTFE may influence the surface chemistry and thermal stability of the film.

Spectral deconvolution of the 2D and D+D' bands was performed using Voigt fitting to resolve overlapping features (Figure 4A (ii)–(iii)). In the EATS‐P4‐3.0 (GO) sample, the 2D and D+D' peaks appeared at 2695 and 2914 cm^−1^, respectively. These downshifts and high D band intensity reflect increased structural disorder and defect density. In contrast, the EATS‐P4‐5.0 (rGO) sample exhibited upshifted 2D and D+D' peaks at 2705 and 2929 cm^−1^, respectively, indicative of partial restoration of sp^2^ domains and reduced oxygenated functionalities, consistent with the increased G/D ratio. Based on the Raman results, contour maps of the G/D ratio and PTFE‐related signal intensity were constructed to visualize distinct tuning of carbon films across various EATS conditions (Figure 4B; Figure S6). The G/D ratio map highlights the tunability of the EATS process, demonstrating precise control over sp^2^/sp^3^ carbon ratios via modulation of power density and duration (Figure 4B). Regions with G/D ratios exceeding 1.2 suggested the formation of high‐quality rGO with well‐defined graphitic frameworks. Simultaneously, the PTFE signal intensity map provided information on the distribution and retention of PTFE within the composite films (Figure S6). Notably, PTFE‐related signal intensity was observed predominantly under higher power or longer duration, implying that extended heating promotes PTFE volatilization and incorporation into the film. This highlights the role of process optimization in tailoring composite properties, either by promoting PTFE retention or its controlled removal to engineer desired surface functionalities. Furthermore, control experiments using PTFE‐free CP yielded negligible Raman signals under EATS‐P4‐7.0 and EATS‐P5‐7.0, confirming that the presence of PTFE is critical for efficient exfoliation and film formation (Figure S7 and Note S1).

To further evaluate the spatial distribution and uniformity of rGO within the synthesized films, large‐area Raman mapping was performed on the EATS‐P4‐5.0 sample, including both an intact rGO region and a mechanically delaminated area created using a razor blade (Figure 4C). The mapping focused on the G, D, and 2D+D′ bands, as well as the G/D intensity ratio, enabling a comprehensive structural analysis across the film surface (Figure 4C (i)). Signal attenuation of all peaks at the delamination boundary confirmed well‐defined rGO film coverage over a large area (Figure 4C (ii)). To further substantiate this observation, a line profile was extracted along a transect from Point A to Point D across the delaminated boundary (Figure 4C (iii)). The raw Raman spectra demonstrate a distinct attenuation of the G, D, and 2D+D′ peaks within the delaminated zone, reinforcing the spatial integrity and uniformity of the rGO film in the intact region. These results confirm that the EATS process enables the controlled, large‐area deposition of rGO with high fidelity, highlighting its potential for scalable fabrication of tunable carbon‐based films.

**FIGURE 5:**
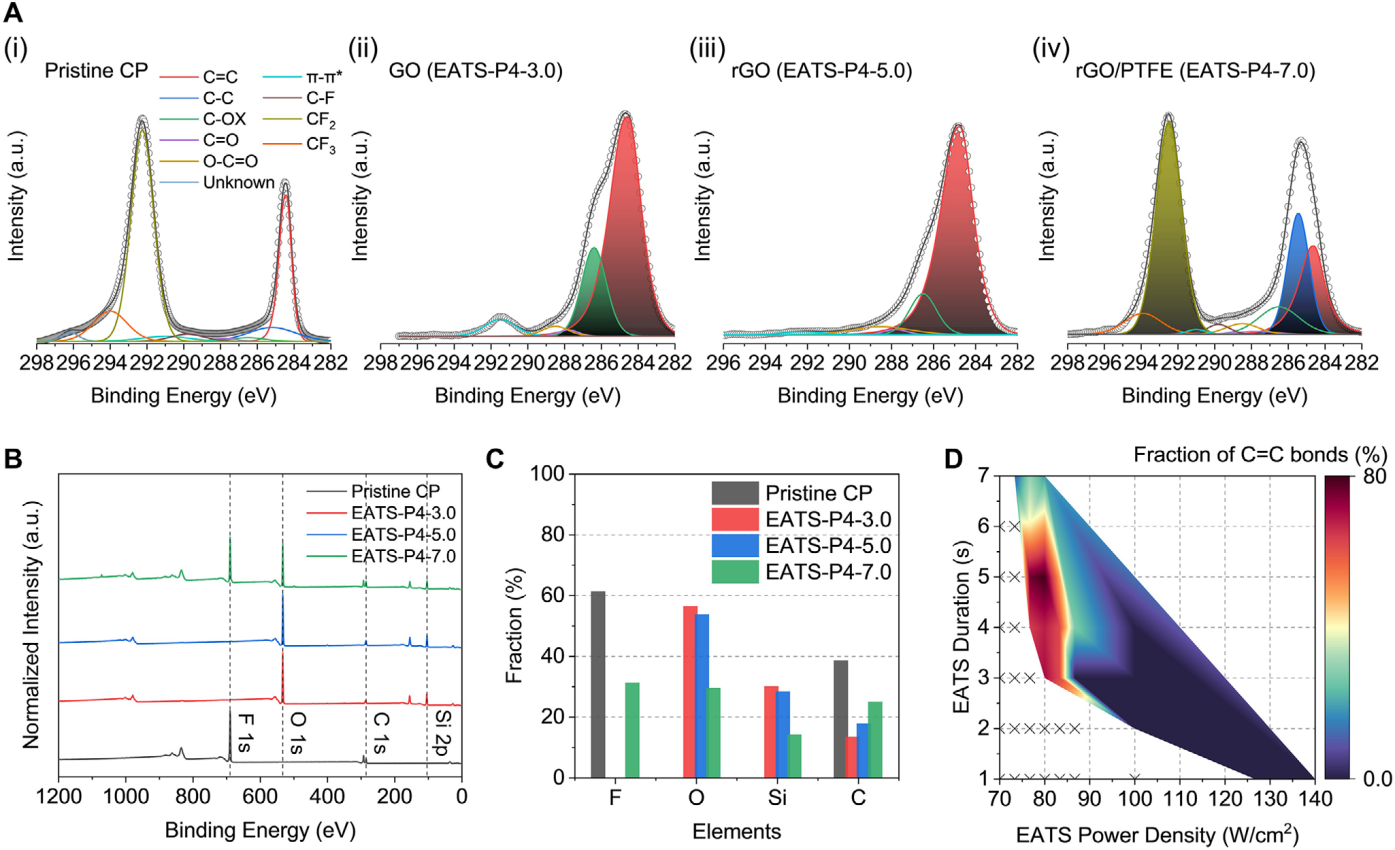
XPS analysis of carbon‐based nanofilms. (A) High‐resolution C 1s spectra of (i) pristine CP, (ii) EATS‐P4‐3.0, (iii) EATS‐P4‐5.0, and (iv) EATS‐P4‐7.0, corresponding to graphite embedded with PTFE, GO, rGO, and rGO/PTFE composite states, respectively. Each spectrum was deconvoluted into chemical states including C = C (∼284.6 eV), C─C (∼285.6 eV), C─OX (∼286.5 eV), C═O (∼287.8 eV), O─C═O (∼288.5 eV), *π–π* ^*^ (∼291.0 eV), C─F (∼289.8 eV), CF_2_ (∼292.0 eV), and CF_3_ (∼294.0 eV). (B) XPS survey spectra of the same samples showing the elemental composition of F, O, Si, and C. (C) Atomic fraction analysis derived from survey spectra, revealing compositional evolution during the EATS process at a fixed power density (P4). (D) Contour map of the sp^2^ C═C bond fraction in C 1s as a function of EATS power density and duration, demonstrating the tunability of rGO content in the resulting composite films. ‘X’ markers indicate conditions where film formation was not observed.

To gain deeper insight into the chemical bonding states and compositional evolution during the EATS process, XPS analysis was performed on pristine CP, EATS‐P4‐3.0 (GO), EATS‐P4‐5.0 (rGO), and EATS‐P4‐7.0 (rGO/PTFE) samples (Figure 5A). In pristine CP, distinct C1s peaks corresponding to sp^2^ C═C (∼284.6 eV) and CF_2_ (∼292.0 eV) bonds were observed, indicating the coexistence of graphitic domains and embedded PTFE (Figure 5A (i); Figures S8 and S9A) [75, 76, 77]. After EATS‐P4‐3.0, the spectrum showed the emergence of oxygen‐containing functional groups, including C─OX (∼286.5 eV), C═O (∼287.8 eV), and O─C═O (∼288.5 eV), suggesting the partial oxidation of graphite into a mildly oxidized GO state (Figure 5A (ii); Figure S9B). The appearance of a pronounced *π–π* ^*^ shake‐up peak (∼291.0 eV) further confirmed the formation of GO. At EATS‐P4‐5.0, oxygen‐related peaks diminished significantly, confirming efficient GO reduction, and the *π–π* ^*^ feature also weakened, suggesting partial graphitic restoration (Figure 5A (iii); Figure S9C). At EATS‐P4‐7.0, strong sp^2^ (C═C) and sp^3^ (C─C, C─F) signals emerged, indicating PTFE retention and formation of hybrid rGO/PTFE nanocomposites (Figure 5A (iv); Figure S9D).

XPS survey spectra revealed significant fluorine content in both pristine CP and EATS‐P4‐7.0 samples, confirming PTFE incorporation, whereas minimal fluorine signals were detected under intermediate processing conditions (Figure 5B,C). In parallel, a slight but consistent decrease in oxygen content was observed from EATS‐P4‐3.0 to EATS‐P4‐7.0, reflecting the gradual reduction of GO to rGO. The overall difference in oxygen atomic fraction between EATS‐P4‐3.0 and EATS‐P4‐5.0 was approximately 2.7 at%, which becomes even smaller when considering the oxygen contribution from the quartz (SiO_2_) substrate. This subtle yet systematic change is consistent with the C 1s analysis and confirms the effective removal of oxygenated functional groups and partial restoration of sp^2^ carbon networks during the EATS process. To visualize the compositional tunability imparted by the EATS process, contour maps of the C═C bond fraction and the fluorine‐to‐carbon (F/C) ratio were constructed under various EATS conditions (Figure 5D; Figure S10). These maps illustrate the systematic modulation of sp^2^ carbon domains and PTFE incorporation as functions of power density and process duration. To further examine the vertical elemental distribution within the rGO/PTFE composite, XPS depth profiling was conducted for the EATS‐P4‐7.0 sample (Figure S11). The F 1s signal gradually decreased with increasing etching time, while the carbon content remained nearly constant and the O 1s signal originated mainly from the exposed quartz substrate. These results indicate that PTFE is primarily localized near the surface, forming a fluorine‐rich top layer over rGO‐rich domains. The observed trends are in excellent agreement with Raman spectroscopy results, both evidencing the restoration of ordered sp^2^ carbon networks, chemical transition pathway, and PTFE‐associated peak intensities. Together, these results confirm that the EATS process enables precise control over the sp^2^/sp^3^ carbon structures and PTFE content, facilitating the fabrication of compositionally tunable carbon‐based nanofilms.

To evaluate process reproducibility, multiple EATS‐P4‐5.0 cycles were performed using a single CP source (Figures S12–S13 and Note S2). Raman spectra across six cycles maintained consistent G/D ratios, indicating minimal structural degradation of the exfoliated rGO (Figure S12). While XPS analysis revealed gradual CF_x_ depletion, suggesting consumption of PTFE over repeated use, the sp^2^ carbon remained stable (Figure S13). These results demonstrate that the EATS process is reproducible and scalable, with the potential for repeated fabrication of high‐quality composite films from a single CP source.

### 2.3 Thickness and In‐Plane Pattern Control of Carbon‐Based Nanofilms

**FIGURE 6:**
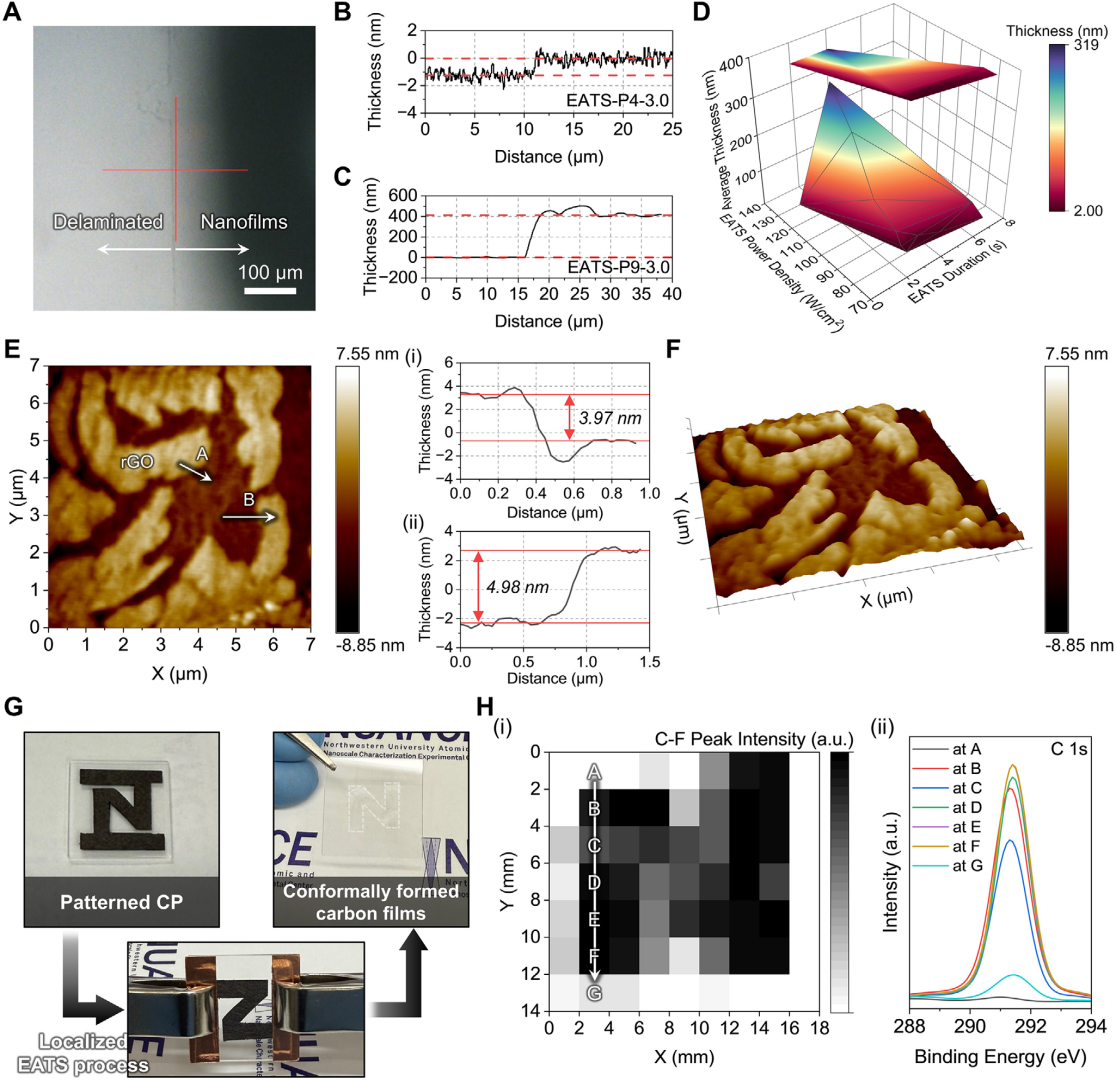
Thickness analysis and in‐plane patterning capability of carbon‐based nanofilms. (A) Optical microscopy image showing the boundary between the nanofilm and the delaminated region, used for thickness measurement. Thickness profiles obtained by surface profilometry for films fabricated under (B) EATS‐P4‐3.0 and (C) EATS‐P9‐3.0 conditions, respectively. (D) 3D contour mapping of average film thickness as a function of EATS power density and duration. (E) AFM surface morphology image of the rGO film (EATS‐P4‐5.0), with corresponding line profiles in (i) A and (ii) B paths. (F) 3D AFM reconstruction of the same film, revealing surface morphology at the nanoscale. (G) Photographs illustrating the localized patterning process using pre‐patterned CP in the shape of an “N” and the resulting conformal film formation via localized EATS. (H) (i) XPS mapping analysis of fluorine distribution in the EATS‐P8‐3.0 sample, based on (ii) the C─F peak intensity in the C 1s spectra at various positions.

The thickness of carbon films synthesized via the EATS process was evaluated using surface profilometry and atomic force microscopy (AFM) to investigate the relationship between process parameters, film thickness, and surface morphology (Figure 6). Thickness was measured across the boundary between the delaminated and intact regions of the film (Figure 6A). Under EATS‐P4‐3.0, the film exhibited a relatively thin layer of ∼1.21 nm, whereas EATS‐P9‐3.0 yielded a significantly thicker film (∼412 nm), attributed to enhanced material deposition at higher power density (Figure 6B,C). As shown in Figure S14, film thickness increased exponentially with power density at a fixed duration of 3.0 s, rising from 2.0 nm at P4 to 318.1 nm at P9. Higher power densities also led to greater variability in thickness, suggesting increased surface roughness under intense thermal conditions. A contour map of film thickness as a function of power density and duration further highlights the excellent tunability enabled by precise control of process parameters (Figure 6D).

AFM imaging provided detailed insight into surface morphology and nanoscale thickness uniformity of the films (Figure 6E,F). A 7 µm × 7 µm area of the EATS‐P4‐5.0 (rGO) film was scanned, revealing few‐layer rGO domains with apparent thickness variations (Figure 6E). Line profiles along paths A and B revealed height differences of ∼3.97 and ∼4.98 nm, respectively, confirming successful deposition of ultrathin rGO films (Figure 6E (i)–(ii)). The corresponding 3D topography showed discrete domains and localized height fluctuations, indicating incomplete surface coverage (Figure 6F). Compared to thicker composite or PTFE‐rich films (>40 nm), these thin rGO layers displayed structural discontinuities and interdomain gaps (Figure S15). While moderate EATS conditions can yield few‐layer rGO, achieving complete uniformity may require further process refinement, such as exploiting the flake texture of CP as a templating scaffold or applying sequential EATS cycles to fill surface voids and enhance layer cohesion.

To demonstrate patterning capability, CP was pre‐shaped into an “N” geometry, enabling localized thermal treatment and spatially selective deposition of films in predefined regions (Figure 6G). Under EATS‐P8‐3.0, a condition that ensures stable rGO/PTFE film formation, the films were conformally deposited along the patterned regions. XPS mapping of the C─F bond intensity, which provides a clear and reliable contrast for visualizing spatial deposition, confirmed localized PTFE incorporation with strong signals detected exclusively within the patterned area (Figure 6H (i)). The corresponding C 1s spectra showed prominent C─F bond peaks, verifying successful PTFE incorporation (Figure 6H (ii)). These results establish the EATS process as a mask‐free, direct‐write patterning technique capable of spatially controlled film deposition defined by thermal stamp geometry. Beyond patterning, the EATS process also enables direct, single‐step fabrication of uniform and large‐area nanofilms under ambient conditions, with precisely tunable thickness controlled by the power density and duration. This simple and scalable approach represents a significant improvement over conventional multi‐step or vacuum‐based fabrication techniques (Figure S16 and Table S1).

### 2.4 Application‐Specific Direct Fabrication of Multifunctional Devices via EATS Process

**FIGURE 7:**
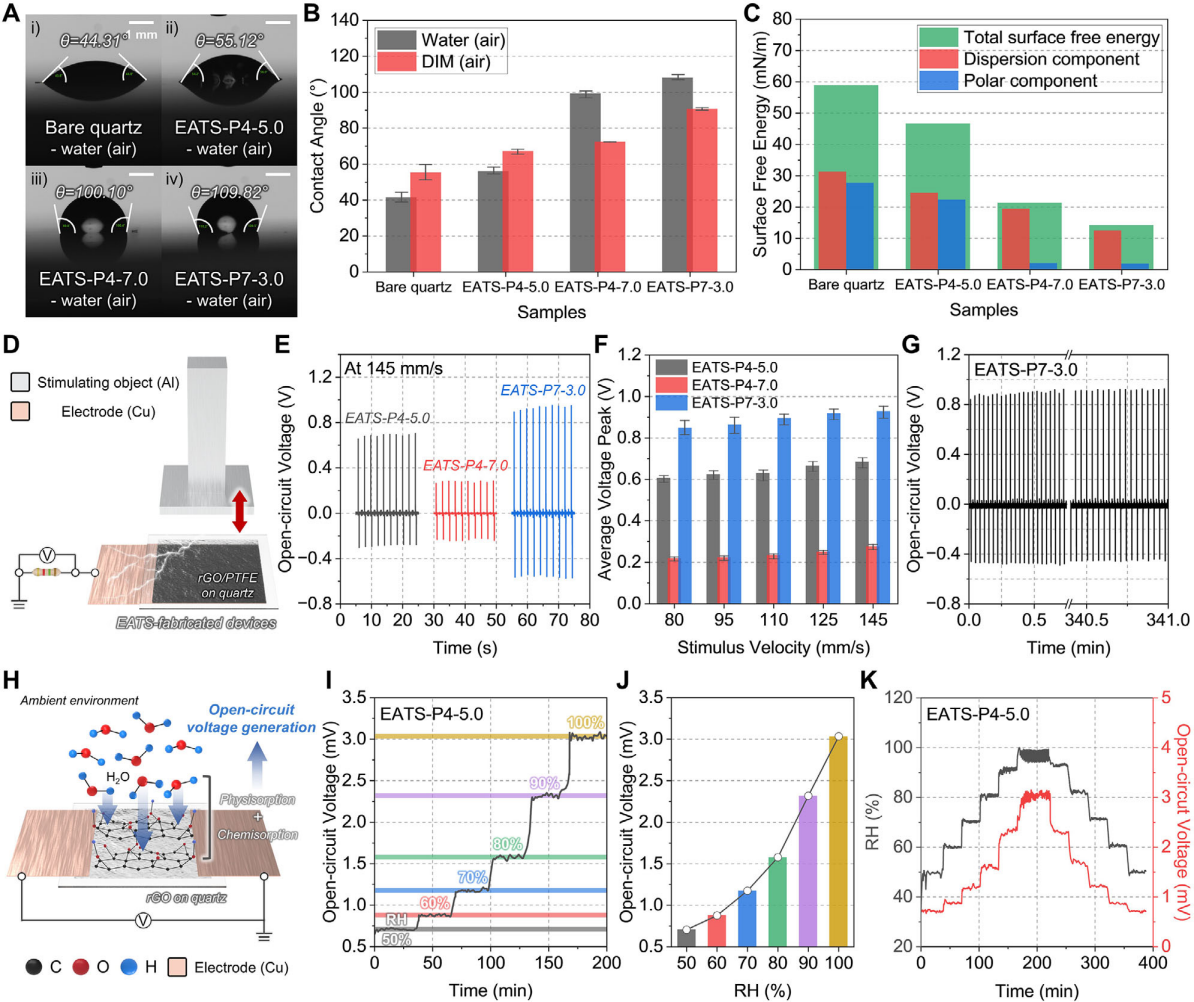
Application‐specific demonstrations of multifunctional devices fabricated via the EATS process. (A) Static contact angle images measured using DI water droplets on (i) bare quartz, (ii) rGO (EATS‐P4‐5.0), (iii) rGO/PTFE (EATS‐P4‐7.0), and (iv) PTFE (EATS‐P7‐3.0). (B) Contact angle measurements using both DI water and DIM droplets, confirming EATS‐enabled tunability of surface wettability across different film types. (C) Surface free energy calculated using the OWRK method. (D) Schematic illustration of the single‐electrode triboelectric nanogenerators (TENG) test setup employing EATS‐fabricated films. (E) OCV profiles of the devices under a stimulus velocity of 145 mm/s. (F) Comparison of average voltage peaks at different stimulus velocities (80–145 mm/s) for the performance tunability of devices fabricated under different EATS conditions. (G) Long‐term electrical stability of the EATS‐P7‐3.0 device over 10 000 cycles (∼5.68 h), demonstrating mechanical durability and signal consistency. (H) Schematic illustration of the humidity sensing mechanism in the EATS‐P4.5.0 device, illustrating water molecule chemisorption and physisorption on the rGO surface, which induces a potential difference across the electrodes. (I) Raw OCV profiles measured at increasing relative humidity (RH) levels from 50% to 100%, showing a distinct stepwise voltage response. (J) Calibration curve of OCV as a function of RH. (K) Hysteresis behavior of the sensor under cyclic RH variation from 50% to 100% and back to 50%, demonstrating reversible signal characteristics.

To demonstrate the practical applicability of EATS‐fabricated carbon films, multifunctional thin‐film devices were developed for hydrophobic coatings, mechano‐electric energy harvesting, and humidity sensing (Figure 7). Hydrophobic surface engineering was first demonstrated through static contact angle measurements on films fabricated under different EATS conditions, including bare quartz, rGO (EATS‐P4‐5.0), rGO/PTFE (EATS‐P4‐7.0), and PTFE (EATS‐P7‐3.0) films (Figure 7A–C). The ability to tailor surface wettability, from mildly hydrophobic rGO to highly hydrophobic PTFE composites, not only imparts functional surface characteristics but also offers significant advantages in applications such as self‐cleaning coatings, anti‐fouling layers, and water‐repellent barriers for wearable or energy devices exposed to humid or wet environments. Bare quartz exhibited a contact angle of 44.31°, consistent with its intrinsically hydrophilic nature (Figure 7A (i)). Upon rGO coating (EATS‐P4‐5.0), the contact angle increased to 55.12°, reflecting a modest enhancement in hydrophobicity attributed to the lower surface energy of rGO (Figure 7A (ii)). Notably, the rGO/PTFE and PTFE films showed significantly larger contact angles of 100.10° and 109.82°, respectively, indicative of strong hydrophobic behavior arising from the incorporation of fluorinated PTFE domains (Figure 7A (iii)–(iv)). To further validate the observed trend, contact angle measurements were also conducted using non‐polar diiodomethane (DIM) droplets (Figure 7B). Across all samples, similar increases in contact angle were observed, from 55.31° on bare quartz to 90.74° on PTFE, supporting the tunability of surface wettability through EATS processing parameters. Based on the measured contact angles for both DI water and DIM, the surface free energy (σ_s_) was calculated using the Owens–Wendt–Rabel–Kaelble (OWRK) model. This method separates the surface energy into dispersive (σ_s_^D^) and polar (σ_s_^P^) components, using the following equation for interfacial tension (σ_sl_):

(7) $$\sigma_{\mathrm{sl}}=\sigma_{s}+\sigma_{l}-2\left(\sqrt{{\sigma_{s}}^{D}\cdot {\sigma_{l}}^{D}}+\sqrt{{\sigma_{s}}^{P}\cdot {\sigma_{l}}^{P}}\right)$$

where σ_l_ is the surface tension of the test liquid, and superscripts D and P denote dispersive and polar contributions, respectively. Young's equation is then applied to relate the interfacial tension to the measured contact angle (θ):

(8) $$\cos\theta =\frac{\sigma_{s}-\sigma_{\mathrm{sl}}}{\sigma_{l}}\;$$

By applying linear regression to data obtained from two liquids of known surface energy components, the polar and dispersive contributions of each solid surface were extracted. As shown in Figure 7C, the total surface energy decreased from bare quartz (∼58.94 mN/m) to PTFE (∼14.13 mN/m), with a pronounced reduction in the polar component and a more gradual decrease in the dispersive component. Particularly, the polar component decreased sharply from 22.24 mN/m for rGO (EATS‐P4‐5.0) to 1.96 mN/m for rGO/PTFE (EATS‐P4‐7.0), highlighting the role of PTFE in significantly lowering surface polarity. This dramatic modulation of surface polarity and energy, achieved purely through EATS parameter tuning, positions the platform as a promising solution for scalable hydrophobic coatings and interfacial engineering. Such controllability, achieved under single‐step and ambient conditions, distinguishes EATS from conventional coating methods that typically rely on multi‐step solvent dispersion, dip or spray coating, followed by thermal sintering, or electrostatic deposition, offering a simpler and more versatile route to uniform and adherent hydrophobic films.

In addition, the EATS‐fabricated films were directly utilized as energy harvesters (Figure 7D–G). TENGs were assembled in a single‐electrode configuration, in which carbon films were deposited on a quartz substrate with a copper electrode (Figure 7D). An aluminum (Al) stimulating object was repeatedly contacted and separated, with a contact area of 1 cm × 1 cm. As shown in Figure 7E, the EATS‐P4‐5.0 device (rGO) generated an average OCV of 0.68 V, and the EATS‐P4‐7.0 device (rGO/PTFE) exhibited a lower OCV of 0.28 V, while the EATS‐P7‐3.0 device (PTFE) yielded the highest OCV of 0.93 V, at a stimulation velocity of 145 mm/s. These differences are attributed to intrinsic triboelectric properties [78]. PTFE, located near the negative end of the triboelectric series, exhibits strong electron affinity and high charge retention, particularly in contact with tribo‐positive Al, thus generating high OCV. In contrast, rGO has moderate triboelectric polarity and limited surface charge retention, resulting in lower output. The rGO/PTFE hybrid exhibited the lowest OCV due to the screening and dissipation of triboelectric charges by the conductive rGO domains, which reduced the effective surface charge density. Additionally, the heterogeneous interface between rGO and PTFE may hinder the formation of a robust triboelectric double layer, thereby diminishing contact electrification and suppressing voltage output.

To further investigate output characteristics, stimulation velocity varied from 80 mm/s to 145 mm/s (Figure 7F; Figure S17). As expected, all devices exhibited increased output with increasing velocity driven by greater contact energy and charge transfer efficiency. The EATS‐P7‐3.0 device consistently delivered the highest and most stable voltage performance, with output increasing from 0.85 V at 80 mm/s to 0.93 V at 145 mm/s, confirming its superior energy conversion capability. For comparison, a TENG device constructed from pristine CP produced only small and unstable voltage signals under identical test conditions, highlighting the clear performance advantages of the EATS‐derived films (Figure S18). In addition, long‐term durability was validated by cycling the EATS‐P7‐3.0 device over 10,000 operations (∼5.68 h) (Figure 7G). The OCV remained stable with only ∼2.8% fluctuation, indicating the mechanical and electrical robustness of the EATS‐fabricated films under repetitive operation. Furthermore, load matching tests revealed a maximum power density of 10.11 mW cm^−3^ at an external resistance of 2.5 MΩ (Figure S19). This performance exceeds the output of many reported triboelectric devices, primarily due to the unique material characteristics imparted by the EATS process (Table S2). Specifically, the PTFE films synthesized via EATS exhibit highly uniform and compact surface morphologies, enhanced fluorine content that boosts negative triboelectric polarity, and superior interfacial adhesion to the substrate through vdW and hydrogen bonding interactions. These synergistic features contribute to reduced charge dissipation, enhanced dielectric polarization, and stable triboelectric contact behavior, collectively resulting in superior energy harvesting performance. These findings establish EATS‐fabricated PTFE films as a promising material platform for advanced triboelectric energy harvesting applications.

Finally, to demonstrate environmental sensing capabilities, humidity sensors were directly fabricated from rGO films (EATS‐P4‐5.0) by attaching copper electrodes to both ends (Figure 7H). The sensing mechanism relies on the interaction of ambient water molecules with oxygen‐containing functional groups (C─OX, C═O, O─C═O) on the rGO surface (Figure 5A (iii)). As RH increases, water molecules are physisorbed onto the rGO surface or form hydrogen bonds (chemisorption) with these polar sites. The adsorbed water molecules, possessing a permanent dipole moment, tend to align directionally along the surface, leading to the formation of a dipole layer and a modulation of the local surface potential [79, 80]. Due to local asymmetry in adsorption, the surface potential becomes asymmetric across the film, resulting in a measurable OCV between the two electrodes. Accordingly, the rGO film (EATS‐P4‐5.0) was chosen as the sensing material because its moderate density of oxygenated sites facilitates dipole‐induced potential generation, while its high electrical conductivity ensures stable and effective signal transduction. Upon stepwise increases in RH from 50% to 100% at 30‐min intervals, the OCV of the sensor exhibited an exponential rise from 0.71 mV to 3.03 mV (Figure 7I; Figure S20). This monotonic response was used to construct a calibration curve for ambient humidity detection (Figure 7J). The increase in OCV is attributed to the cumulative formation of aligned surface dipoles, as physisorbed water molecules progressively occupy oxygen‐functionalized sites on the rGO surface. At higher RH levels, both the density and orientation of these dipoles increase, amplifying the surface potential gradient across the electrodes. To evaluate sensing stability, a hysteresis test was performed by cycling RH from 50% to 100% and back to 50% (Figure 7K). The OCV profile displayed clear and reversible stepwise transitions, confirming the repeatability and stability of the sensing mechanism under ambient conditions. Notably, the average deviation from reference RH values measured by a commercial humidity sensor was less than 3.56%, confirming the high accuracy and reliability of the EATS‐fabricated rGO humidity sensor (Figure S21). Furthermore, based on the dynamic response profiles in Figure 7I,J, the device exhibited an average sensitivity of 0.47 mV/RH% and an average response time of 5.75 s, demonstrating rapid and reliable humidity detection enabled by the rGO films (Figure S22 and Note S3).

In comparison, additional humidity‐sensing tests were conducted for EATS‐P4‐7.0 (rGO/PTFE) and EATS‐P7‐3.0 (PTFE) devices (Figure S23). The rGO/PTFE‐based device showed irregular and fluctuating OCV responses with reduced amplitude, which can be attributed to the heterogeneous distribution of conductive rGO and insulating PTFE domains that disrupt uniform charge transport and water adsorption. In contrast, the PTFE‐based device exhibited a nearly flat OCV response due to its high hydrophobicity and electrical insulation, which severely limit dipole formation and potential buildup. These results confirm that humidity‐sensing performance strongly depends on the sp^2^/sp^3^ carbon ratio and surface polarity, and that the rGO film (EATS‐P4‐5.0) provides the optimal balance between conductivity and hydrophilicity for stable and sensitive response behavior. Beyond humidity sensing, the EATS‐fabricated films could also be conformally transferred onto various target substrates, such as silicon wafers or flexible polyimide films, using a thermal release tape‐assisted method (Figures S24 and S25). This substrate versatility broadens the applicability of the carbon‐based nanofilms to a wide range of energy devices.

These results powerfully validate the multifunctional potential of the EATS platform. By simply adjusting power density and duration, films with tailored surface energy, triboelectric polarity, or functional groups can be directly deposited onto a substrate, eliminating the need for transfer, post‐treatment, or masking. This capacity to fabricate structurally distinct, application‐ready devices, from hydrophobic surfaces to energy harvesters and humidity sensors, using a single, scalable method illustrates the profound utility of EATS in wearable electronics, environmental monitoring, and energy‐autonomous systems.

## 3 Conclusion

In this study, we developed a single‐step EATS method that enables the direct, scalable, and lithography‐free fabrication of multifunctional carbon‐based nanofilms. By applying localized Joule heating to PTFE‐embedded CP under ambient conditions, the EATS process simultaneously induces graphite exfoliation, GO reduction, and PTFE incorporation, eliminating the need for multi‐step or vacuum‐based processing. Key film characteristics, including thickness, surface morphology, and chemical composition, were precisely tuned by controlling the power density and process duration. Comprehensive physicochemical analyses confirmed the formation of hybrid structures with modulated sp^2^/sp^3^ carbon ratios and fluorine content. Notably, the EATS process enables spatially selective film deposition defined by stamp geometry, without requiring any lithographic techniques. The applicability of this platform was demonstrated through the direct fabrication of three representative devices. Hydrophobic coatings with tunable contact angles from 44.3° (bare quartz) to 109.8° (PTFE‐rich film) and reduced surface energy down from 58.9 mN/m to 14.1 mN/m were achieved by adjusting the rGO/PTFE composition. TENGs fabricated via EATS delivered high power density of 10.11 mW cm^−3^ and exhibited long‐term durability over 10 000 operation cycles with less than 2.8% output variation. Humidity sensors based on EATS‐fabricated rGO films showed reliable and reversible voltage responses across a wide RH range (50–100%) with a low sensing error of 3.56%, confirming their accuracy and environmental stability. These results collectively establish EATS as a versatile and generalizable fabrication strategy for multifunctional nanocomposite films and device platforms. Its simplicity, tunability, and direct integration capability offer a transformative alternative to conventional fabrication approaches, with strong potential for applications in flexible electronics, energy harvesting, and environmental monitoring.

## 4 Experimental Section

### 4.1 EATS Process for Tunable Carbon‐Based Nanofilms

Carbon paper (Fuel Cell Store, Toray Carbon Paper 060, 5 wt.% PTFE content, thickness ∼190 µm, electrical resistivity ∼5.8 mΩ·cm, thermal conductivity ∼21–23 W/m·K, porosity ∼78%, USA) was placed onto a quartz substrate (Fisher Scientific, thickness ∼1/16 inch, USA) pre‐cleaned with acetone and DI water. Copper tapes were attached to both ends of the CP, and electrical alligator clips were connected to the tapes to apply power using a programmable power supply (Chroma, 62060D‐100, USA). Power densities were varied from 70 to 140 W/cm^2^ and process durations ranged from 1.0 to 7.0 s. After cooling for 1 min, films were rinsed with ethanol. Thermal response and heat distribution during EATS were monitored using an IR camera (FLIR, A6255SC). TGA–MS (NFEC‐2021‐04‐270025) was performed using analyzers (NETZSCH TG 209 F1 and NETZSCH STA 409 PC, Germany) interfaced with a gas chromatograph–mass spectrometer (Agilent 8890 GC / 5977B MS, USA).

All EATS experiments involving PTFE should be conducted in a well‐ventilated fume hood equipped with proper exhaust treatment to safely remove any potential fluorine‐containing decomposition gases, such as hydrogen fluoride (HF). Operators should wear appropriate personal protective equipment, including nitrile gloves, lab coats, face shields, and safety goggles, throughout the experiments. All PTFE residues and processed substrates must be collected in sealed containers and disposed of in accordance with chemical waste management and environmental safety regulations.

### 4.2 Characterization of Films

Raman spectroscopy (Horiba LabRAM system) was used to investigate the structural features of carbon species and PTFE. X‐ray photoelectron spectroscopy (Thermo Fisher, NEXSA G2) was used to analyze chemical state analysis of carbon, oxygen, and fluorine. Scanning electron microscopy (Hitachi SU8030 and SU8700) and energy dispersive spectrometry (Oxford, Ultim Max) were employed to analyze surface morphology and elemental distribution. Samples were sputter‐coated with 1 nm Au/Pd prior to imaging. Elemental maps were processed using ImageJ software, where the mean pixel intensities were extracted after background subtraction and normalization to the total signal intensity. The F/C ratios were calculated as normalized (F_mean_/C_mean_) values for quantitative comparison between regions. Film thickness was measured via surface profilometry (Veeco, Dektak 150) using step height across a razor‐blade‐scratched region. Atomic force microscopy (Bruker, FastScan) used for nanoscale morphology and thickness data.

### 4.3 Direct Fabrication of Application Devices

For transferring the films to other substrates, polymethyl methacrylate (PMMA; Sigma–Aldrich, 15 kg/mol) was dissolved in toluene at 10 wt.% and spin‐coated onto the fabricated films at 500 rpm for 10 s and 5000 rpm for 45 s. The films were baked at 100 °C for 10 min, followed by application of thermal release tape on top. The PMMA/carbon films stack was then detached, placed onto a target substrate, and heated at 120 °C for 15 min. Upon tape removal and acetone rinsing for 20 min, the PMMA was dissolved, leaving the carbon film conformally transferred. Hydrophobic coating devices were fabricated by directly depositing rGO, rGO/PTFE, and PTFE films onto substrates under EATS‐P4‐5.0, P4‐7.0, and P7‐3.0 conditions, respectively. TENG devices were similarly prepared by applying copper tape to the underside of each quartz substrate as a single electrode, forming operational triboelectric devices. For humidity sensors, copper electrodes with 10 mm spacing were attached to both ends of the rGO film synthesized under EATS‐P4‐5.0 conditions.

### 4.4 Performance Evaluation of Application Devices

Hydrophobicity was evaluated by dispensing 1–2.5 µL droplets of DI water and DIM onto bare quartz and nanofilm‐coated surfaces. Static contact angles were measured using a contact angle goniometer (Kruss, DSA 25). Surface free energy was calculated using the OWRK method, which separates total surface energy into polar and dispersive components based on contact angle data with both polar and nonpolar liquids. TENG device performance was assessed using a programmable XYZ linear actuator (Chongqing UMot Technology; maximum speed: 200 mm/s, maximum acceleration: 3000 mm/s^2^, accuracy: 50 µm, China). A 1 cm × 1 cm aluminum‐coated 3D‐printed stimulator was used for periodic contact–separation motion. Actuation velocities ranged from 80 to 145 mm/s. Output signals were acquired using a digital oscilloscope (Tektronix, DPO2004B) and a multimeter (Keysight, 34401A). OCV and output voltages were recorded under external resistances of 100 kΩ to 10 MΩ at a velocity of 145 mm/s. Durability was evaluated through continuous operation for 10 000 cycles under identical conditions, and output fluctuation was monitored. Humidity sensing performance was measured in a programmable temperature–humidity chamber (Espec, SH‐642, USA). RH was systematically varied from 50% to 100% at 298.15 K, while transient RH profiles were logged using a data logger (OMEGA Engineering, HH314A, Korea). OCV responses of the sensors were monitored in real time using a digital multimeter and oscilloscope.

## Author Contributions

Conceptualization was carried out by B.S.; methodology was developed by B.S., Y.C., G.S.S., M.S., K.K., X.H., and J.K.; investigation was performed by B.S. and Y.C.; the original draft was written by B.S. and Y.C.; review and editing of the manuscript, as well as funding acquisition and supervision, were conducted by W.C., X.C., and V.P.D.

## Conflicts of Interest

The authors declare no conflict of interest.

## Supporting information




**Supporting file**: adma72116‐sup‐0001‐SuppMat.docx

**Supporting file**: adma72116‐sup‐0002‐VideoS1‐S4.mp4

## Data Availability

The data that support the findings of this study are available from the corresponding author upon reasonable request.
