# Supplementary material for: Electrically Assisted Thermal Stamping of Tunable Carbon‐Based Nanofilms for Direct Fabrication of Hydrophobic, Energy Harvesting, and Sensing Devices
Source: Adv Mater. 2026 Jan 8;38(27):e16478. doi: 10.1002/adma.202516478 (PMC13173400; doi:10.1002/adma.202516478)
Supplement: Supplementary file 1 — Supporting file: adma72116‐sup‐0001‐SuppMat.docx [file ADMA-38-e16478-s002.docx]

**[Supplementary Information]**

**
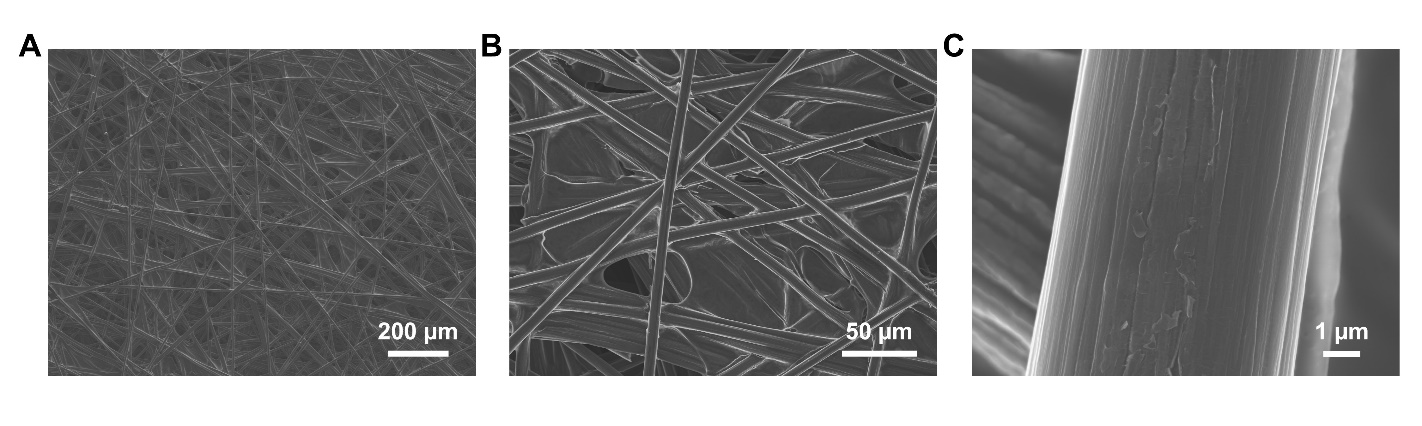
**

**Figure S1. SEM image of pristine carbon paper (CP) showing a porous carbon fiber network structure with polytetrafluoroethylene (PTFE) (~5 wt%), captured at magnifications of (A) ×80, (B) ×400, and (C) ×10,000.** This composite architecture serves as both the thermal conductor and precursor source for the EATS process, providing graphite for reduced graphene oxide (rGO) formation and PTFE for fluorinated species incorporation during nanocomposite synthesis.


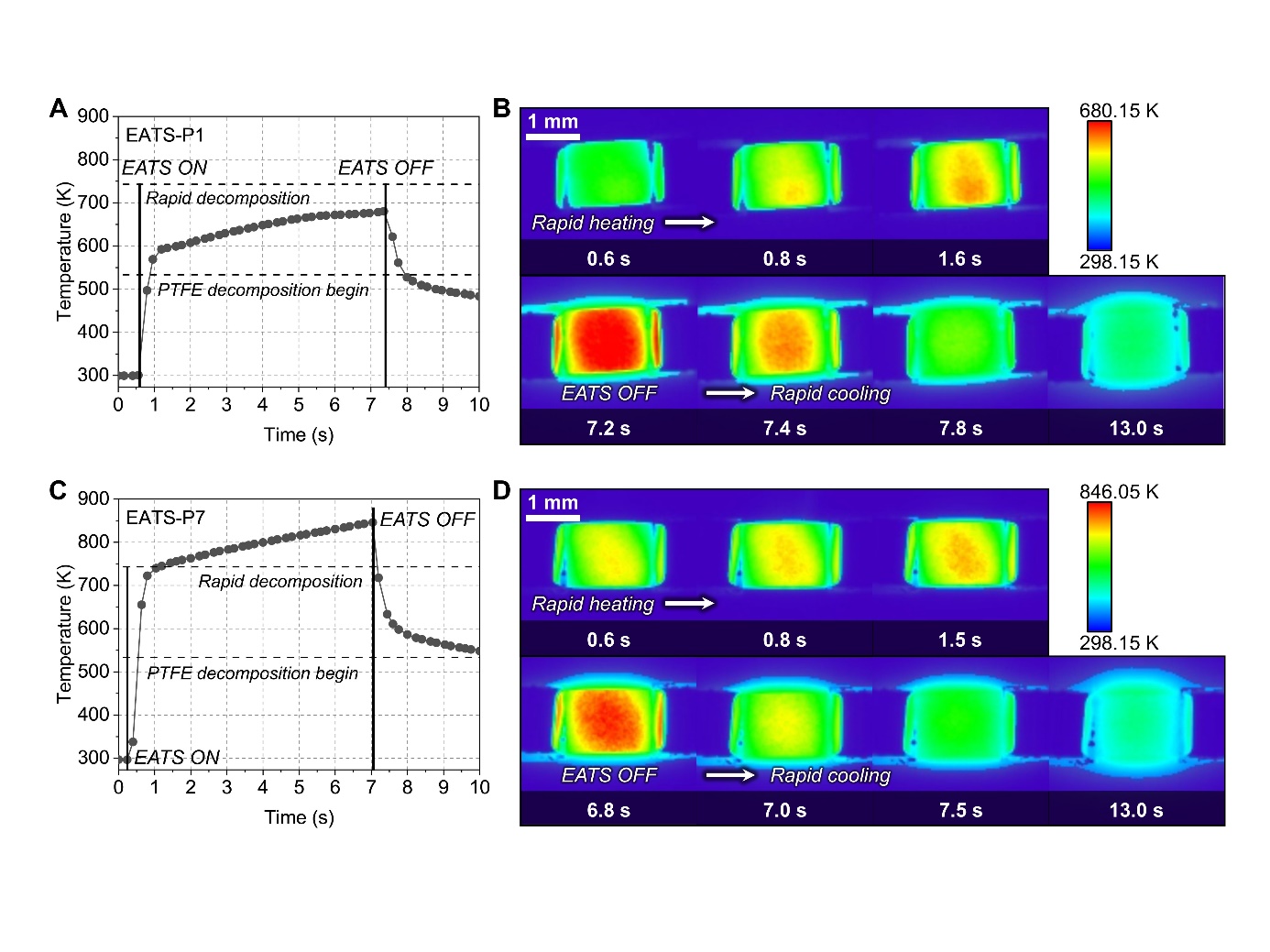


**Figure S2. Temperature profiles and thermal imaging during electrically assisted thermal stamping (EATS) processes.** (A) Temporal temperature profile measured under the EATS-P1 condition for 6.80 s duration. (B) Infrared thermal images showing the spatiotemporal heat distribution during the EATS-P1 process. (C) Temporal temperature profile measured under the EATS-P7 condition for 6.80 s duration. (D) Infrared thermal images showing the spatiotemporal heat distribution during the EATS-P7 process. The uniform and rapid in-plane temperature evolution captured in these measurements reflects the homogeneous Joule-heating environment that drives PTFE decomposition and gas-assisted exfoliation, consistent with the thermal thresholds identified by thermogravimetric analysis–mass spectrometry (TGA–MS) and the delamination conditions predicted by threshold-energy modeling.


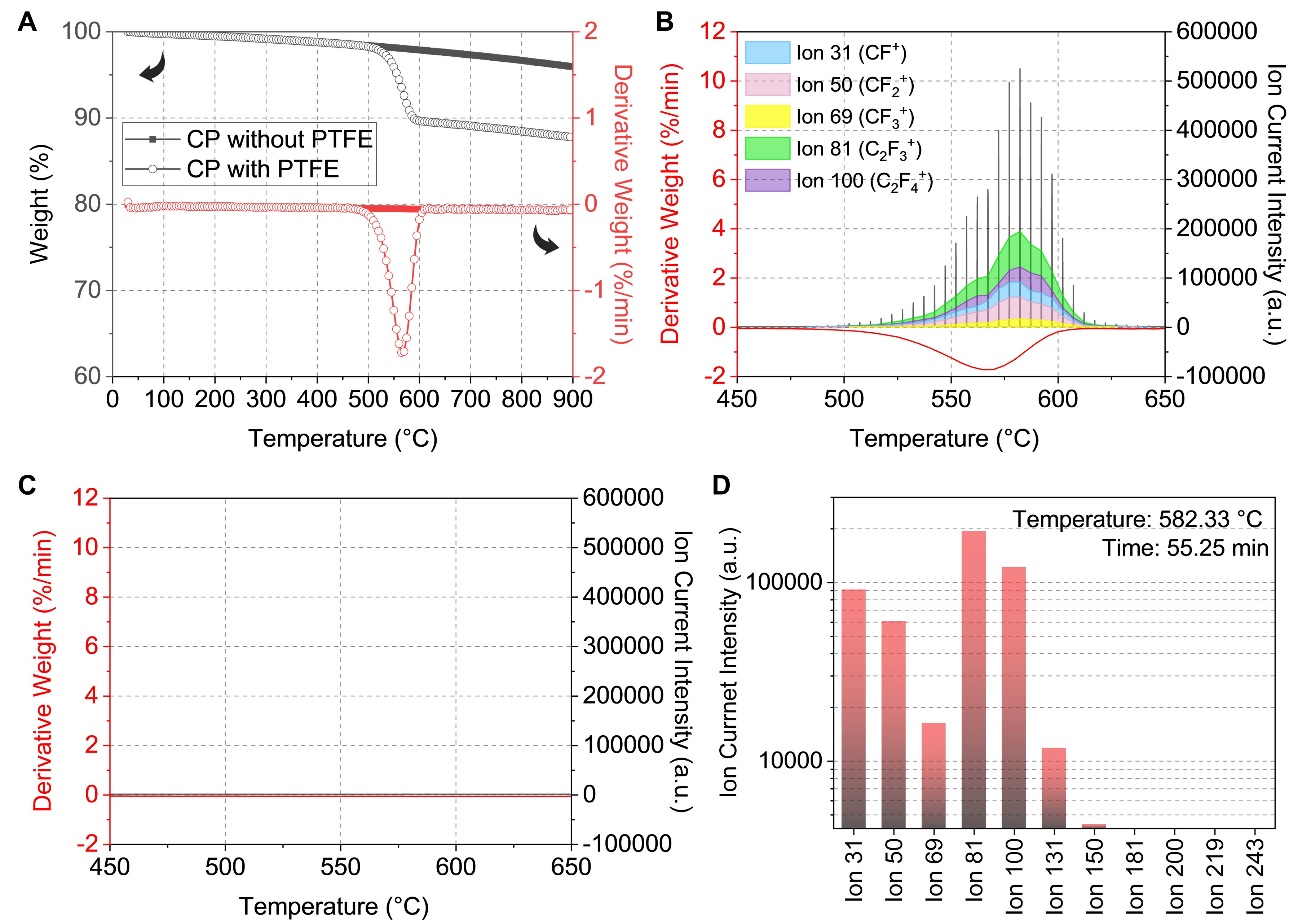


**Figure S3. Thermogravimetric analysis (TGA)–derivative thermogravimetry (DTG) and mass spectrometry (MS) analysis of CP with and without PTFE.** (A) TGA and DTG curves of CP containing PTFE and PTFE-free CP, showing a distinct mass-loss event only in the PTFE-containing sample. (B) MS ion current profiles of characteristic PTFE decomposition fragments detected during the thermal event in CP with PTFE, plotted together with the corresponding DTG signal. (C) DTG and MS profiles of PTFE-free CP, confirming the absence of PTFE-related mass-loss features and fluorocarbon fragments. (D) Representative mass spectrum acquired at the DTG peak temperature (582.33 °C) for CP with PTFE, highlighting dominant fluorocarbon ion species associated with PTFE pyrolysis.

**
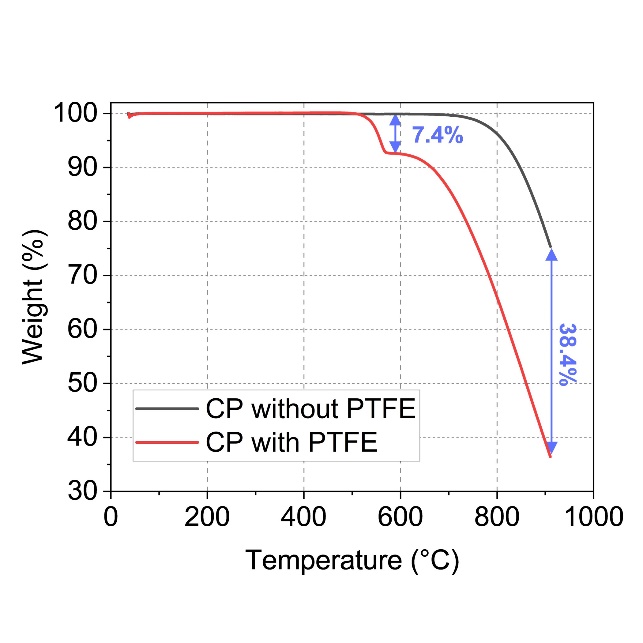
**

**Figure S4. TGA profiles of CP with and without PTFE measured under purified air conditions.** The PTFE-containing CP exhibits an earlier onset of mass loss and a substantially lower residual weight at high temperatures. The indicated 38.4% difference represents the additional mass loss of the PTFE-containing CP relative to PTFE-free CP, reflecting the combined contribution of PTFE volatilization and PTFE-induced oxidation/etching of the carbon framework. The smaller mass-loss feature (~7.4%) at intermediate temperatures corresponds to the initial decomposition of PTFE.


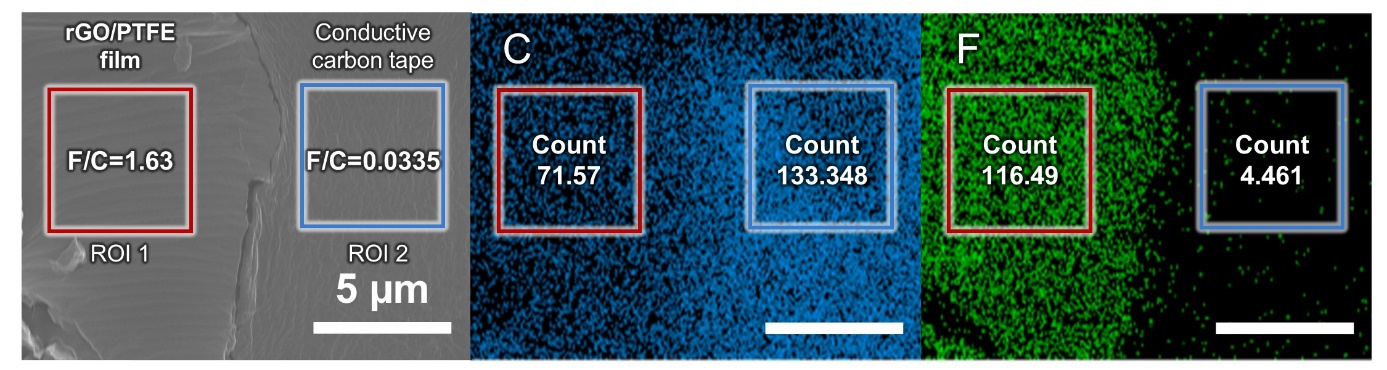


**Figure S5. Quantitative F/C ratio analysis from EDS mapping of the rGO/PTFE film and carbon tape substrate.** An SEM image shows two regions of interest (ROIs) selected on the rGO/PTFE film and the conductive carbon tape. The corresponding C and F elemental maps were analyzed using ImageJ software, where integrated intensity counts were measured within identical ROIs. The F/C ratio was calculated as (F count / C count), confirming strong fluorine enrichment in the rGO/PTFE region (F/C~1.63) compared with the carbon tape (F/C~0.0335). It is noted that this mapping-based ratio is intended for relative, visualization-oriented comparison under identical acquisition conditions, rather than as an absolute atomic ratio, given the known limitations of SEM–EDS quantification and intensity-based calculations for light elements and thin, heterogeneous films.

**
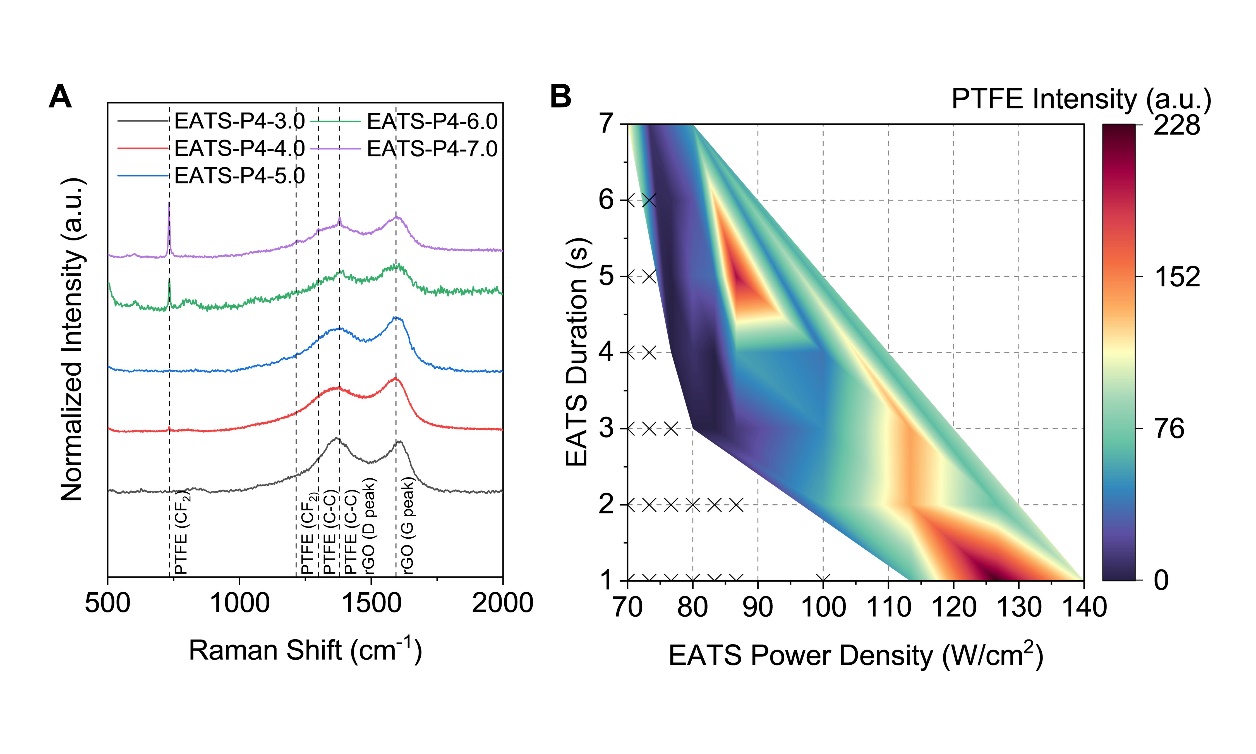
**

**Figure S6. Raman analysis of rGO/PTFE composites synthesized under different EATS conditions.** (A) Raman spectra of EATS-P4 samples with durations ranging from 3 to 7 seconds, showing the spectral evolution corresponding to GO, rGO, and rGO/PTFE composite formation. (B) Contour map of PTFE-related Raman peak (~731 cm^-1^) intensity as a function of EATS power density and duration, indicating compositional tunability via EATS parameters.

**
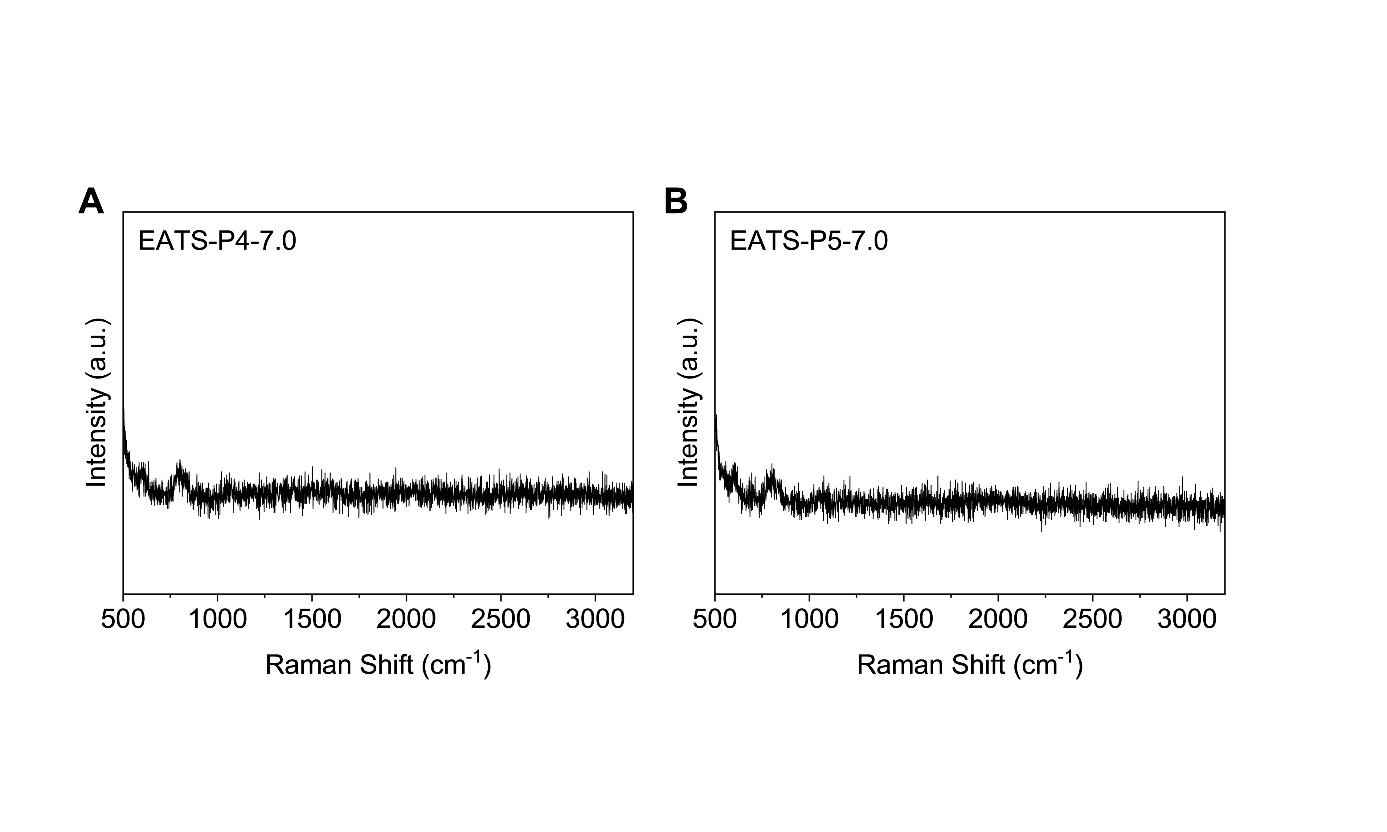
**

**Figure S7. Raman analysis of rGO/PTFE composites synthesized using CP without embedded PTFE.** Raman spectra of (A) EATS-P4-7.0 and (B) EATS-P5-7.0 samples with minimal Raman signals, indicating insufficient graphite exfoliation. These results highlight the essential role of PTFE in promoting interlayer expansion and effective exfoliation during the EATS process.

**Note S1. Correlation between PTFE decomposition, temperature evolution, and nanofilm formation.**

The experimental results provide direct evidence for the synergistic coupling between thermal energy and PTFE-assisted mechanical pressure during the EATS process. As shown in Figure S6, control experiments performed using PTFE-free CP did not yield any detectable nanofilm formation under identical EATS conditions, confirming that the presence of PTFE, and thus the gas pressure generated by its thermal decomposition, is essential for effective exfoliation.

The temporal temperature profiles shown in Figure S2 and Figure 2E reveal that the onset of PTFE rapid decomposition (~673 K) coincides precisely with the initiation of film formation observed in Figure 4B. Under EATS-P1, the surface temperature reaches this decomposition threshold only after approximately 5.80 s, and no film formation is observed prior to that point. In contrast, under EATS-P4 and EATS-P7, the decomposition threshold is achieved within ~2.34 s and ~0.44 s, respectively, which aligns with the appearance of deposited films. This temporal correlation indicates that the volatile species (e.g., CF_2_ and C_2_F_4_) evolved from PTFE decomposition provide localized mechanical expansion and interlayer delamination forces that complement the thermally driven exfoliation of graphite.

This synergy effectively reduces the required thermal energy for exfoliation, enabling film formation at temperatures significantly lower than the theoretical threshold (~1314 K). Operating within this moderate temperature regime minimizes thermal stress while preserving the graphitic lattice integrity and surface uniformity of the resulting nanofilms, thereby improving process stability and reproducibility across multiple EATS cycles.

These results confirm that the EATS mechanism arises from the combined action of thermal excitation and PTFE-induced gas pressure, which jointly facilitate controlled graphite exfoliation and nanofilm deposition under ambient conditions.

**
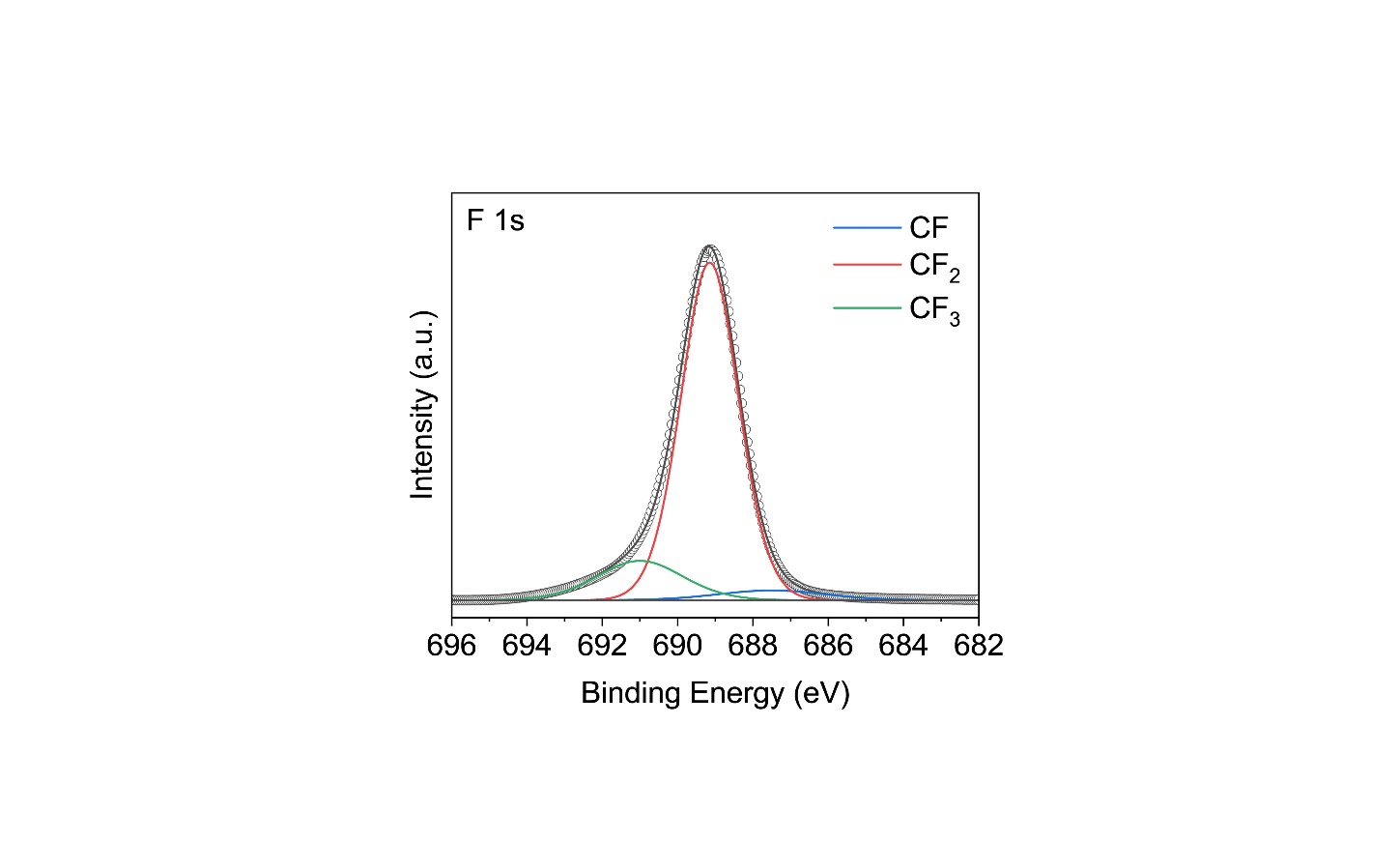
**

**Figure S8. X-ray photoelectron spectroscopy (XPS) F 1s spectrum of pristine CP containing PTFE.** The spectrum is deconvoluted into three components corresponding to CF (~687.5 eV), CF_2_ (~689.2 eV), and CF_3_ (~691.0 eV), confirming the presence of various fluorocarbon bonding states characteristic of PTFE.

**
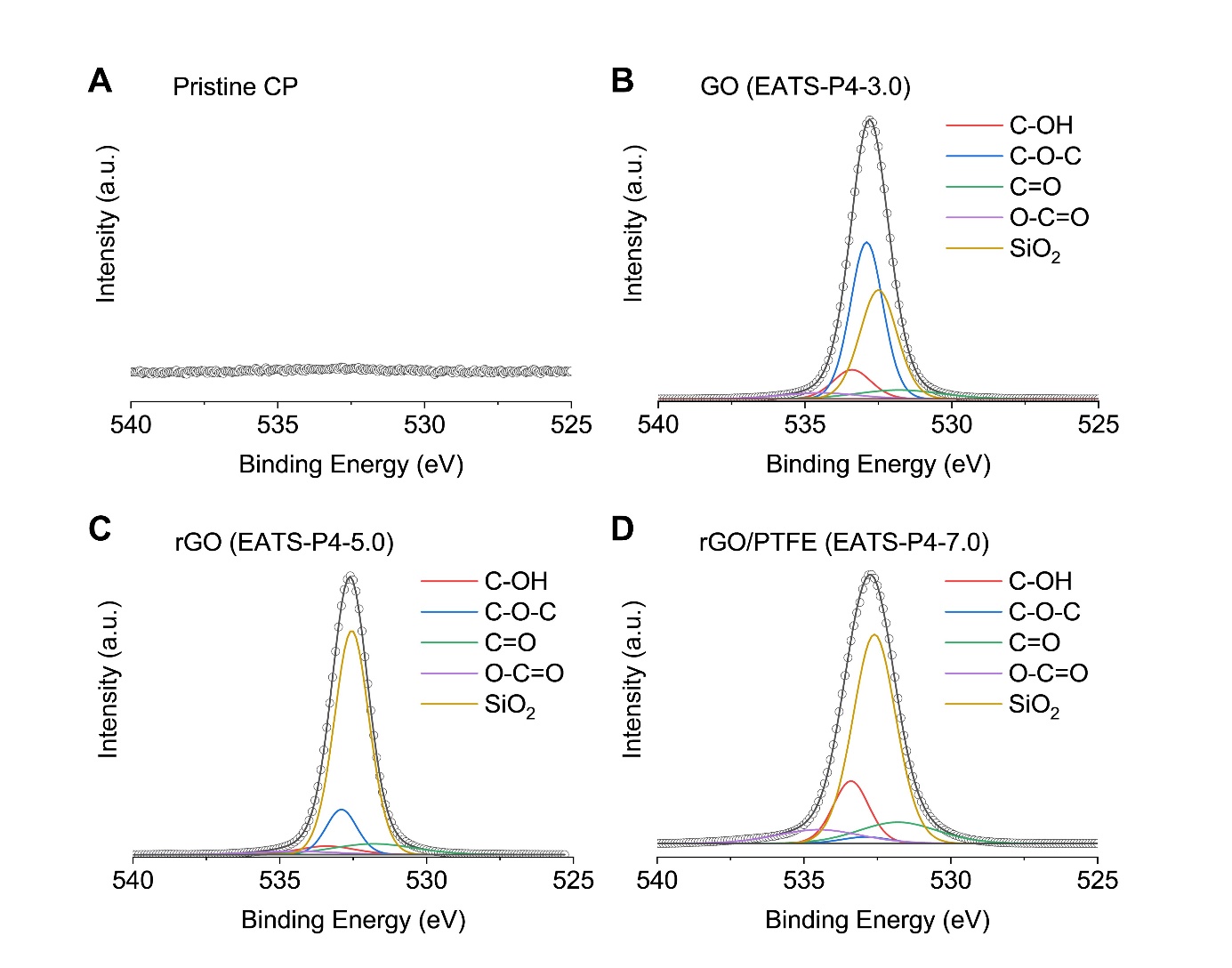
**

**Figure S9. XPS O 1s spectra of (A) pristine CP, (B) EATS-P4-3.0, (C) EATS-P4-5.0, and (D) EATS-P4-7.0, corresponding to graphite embedded with PTFE, GO, rGO, and rGO/PTFE composite states, respectively.** The spectra are deconvoluted into individual oxygen-containing functional groups, including C–OH (~533.4 eV), C–O–C (~532.9 eV), C=O (~531.8 eV), O–C=O (~534.5 eV), and SiO_2_ (~532.5 eV).

**
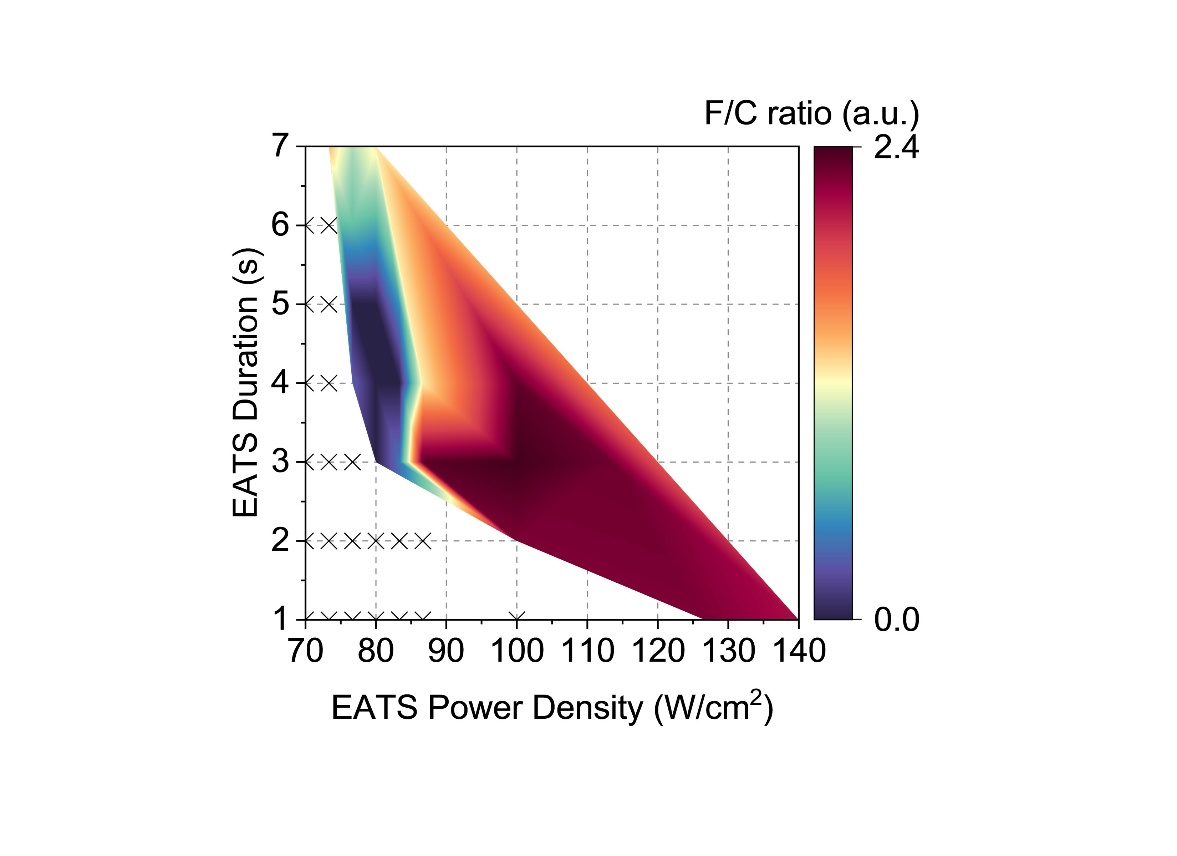
**

**Figure S10. Contour map of the fluorine-to-carbon (F/C) ratio obtained from XPS survey scans under different EATS power densities and durations.** ‘X’ markers indicate conditions where rGO/PTFE film formation was not achieved.


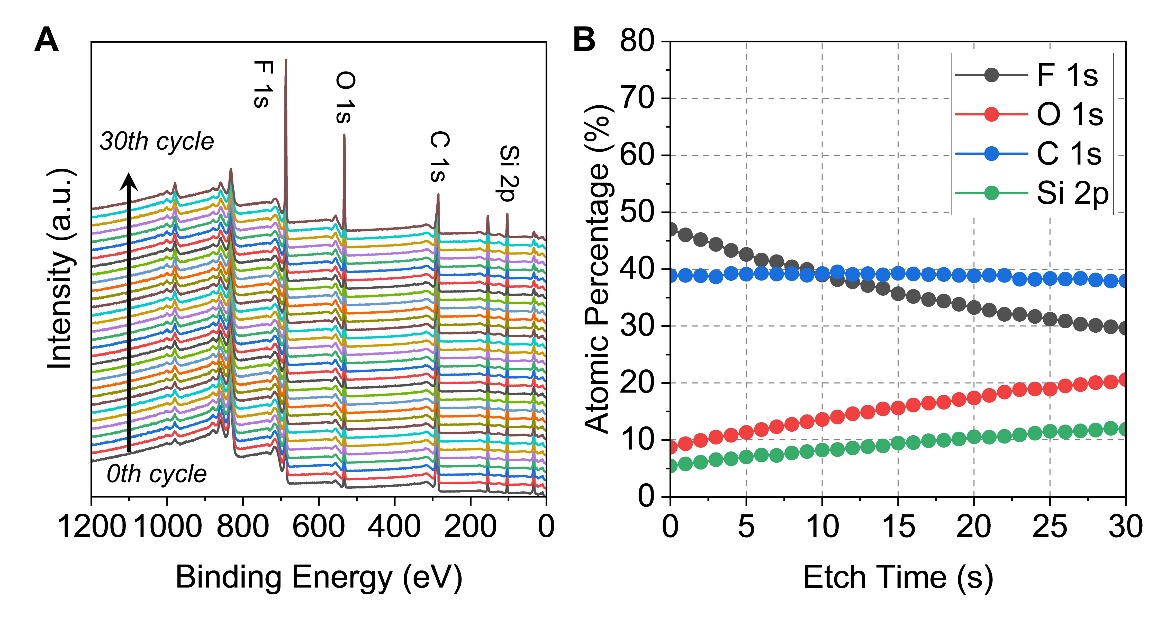


**Figure S11. XPS depth profiling of the EATS-P4-7.0 (rGO/PTFE) sample.** (A) Sequential XPS survey spectra acquired from the 0th to 30th etching cycle, with each cycle corresponding to 1 s of Ar^+^ ion etching at 2000 eV. (B) Evolution of atomic concentrations of F 1s, O 1s, C 1s, and Si 2p as a function of cumulative etching time.

**
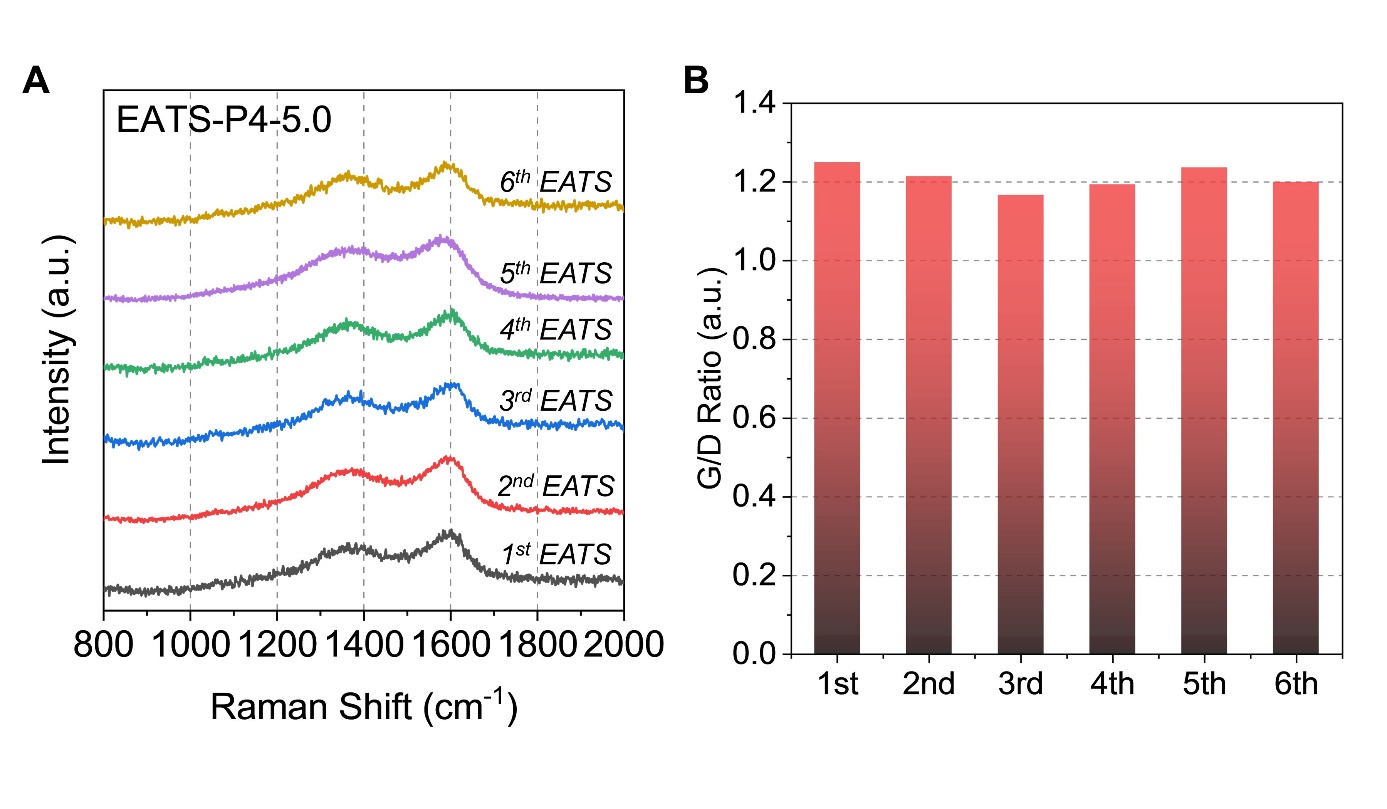
**

**Figure S12. Reproducibility test of rGO/PTFE film fabrication using a single CP over multiple EATS cycles.** (A) Raman spectra of films produced under the EATS-P4-5.0 condition from the 1st to the 6th cycle. (B) Corresponding G/D ratios, showing consistent features across repeated uses.

**
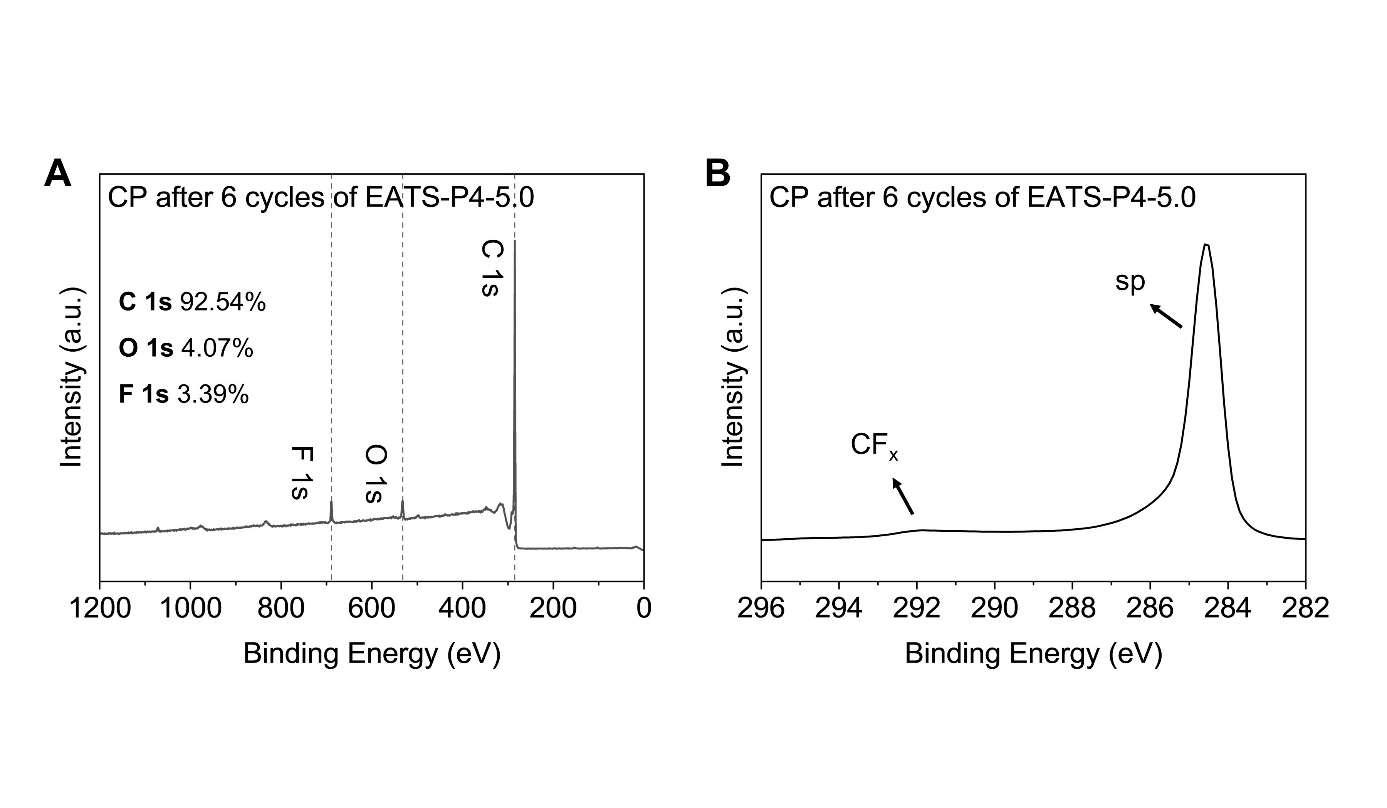
**

**Figure S13. Reproducibility test of rGO/PTFE film fabrication using a single CP over 6 cycles.** (A) XPS survey spectrum of the CP after 6 cycles under the EATS-P4-5.0 condition, showing elemental composition (C 92.54%, O 4.07%, F 3.39%). (B) High-resolution C 1s spectrum of the same sample, indicating the presence of residual CF_x_ species and predominant sp^2^ carbon. These results confirm that PTFE was progressively consumed to facilitate effective exfoliation during repeated EATS processing.

**Note S2. Estimation of PTFE consumption during EATS.**

To estimate the extent of PTFE consumption during the EATS process, TGA and compositional analysis were performed. As shown in Figure S4, CP containing 5 wt% PTFE exhibits a significantly larger mass loss than PTFE-free CP upon heating to 900 °C under air-zero conditions. Given the low initial PTFE loading, this additional mass loss indicates that PTFE is predominantly converted into volatile species, while the remaining mass loss arises from partial oxidation or etching of the graphite framework.

The consumption of PTFE under actual EATS conditions was further evaluated using XPS. Pristine CP shows an initial fluorine content of ~60% (Figure 5B), which decreases to ~3.4% after six EATS cycles under the EATS-P4-5.0 condition (Figure S13). This corresponds to an overall fluorine depletion of ~94%, or an average PTFE consumption of approximately [(60 − 3.4) / 60] / 6 × 100 ≈ 15.7% per EATS cycle, calculated from the cumulative decrease in fluorine content. This value represents an average estimate over multiple cycles.

**
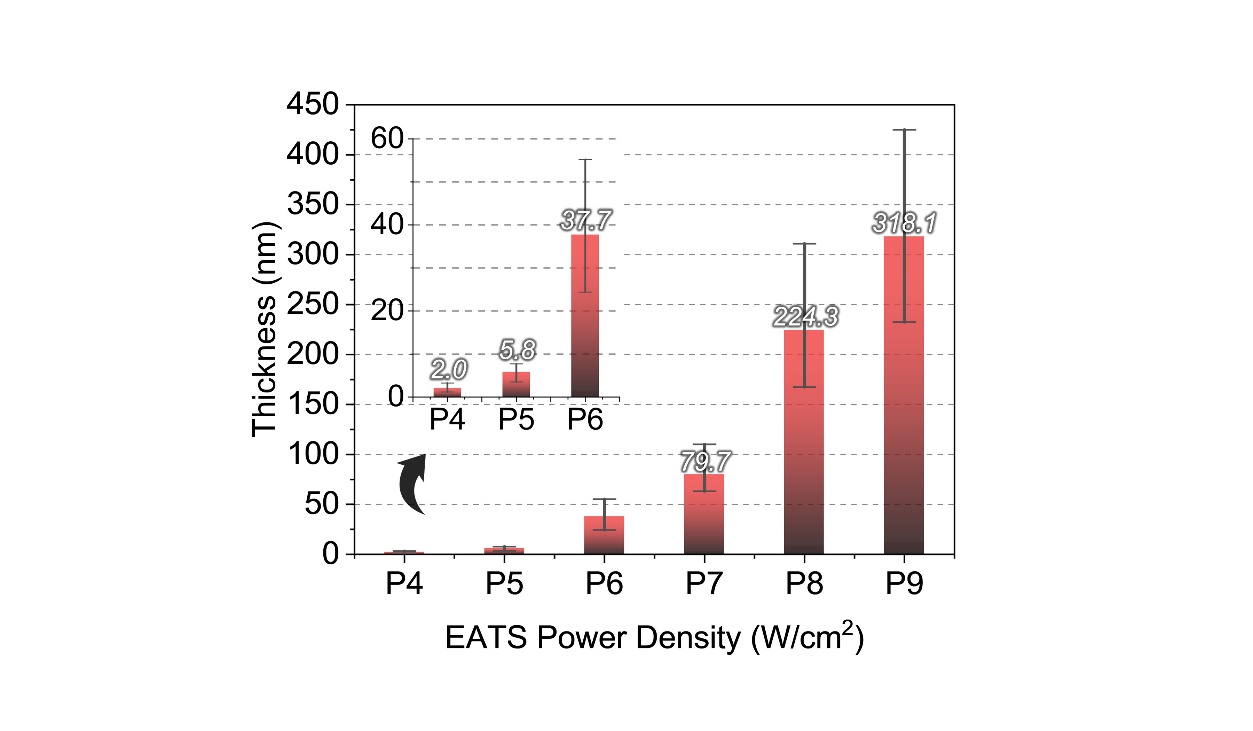
**

**Figure S14. Thickness measurements of rGO/PTFE films fabricated under different EATS power densities (P4 to P9) with a fixed duration of 3 seconds.** Film thickness was measured using a surface profilometer, showing a strong dependence on power density.

**
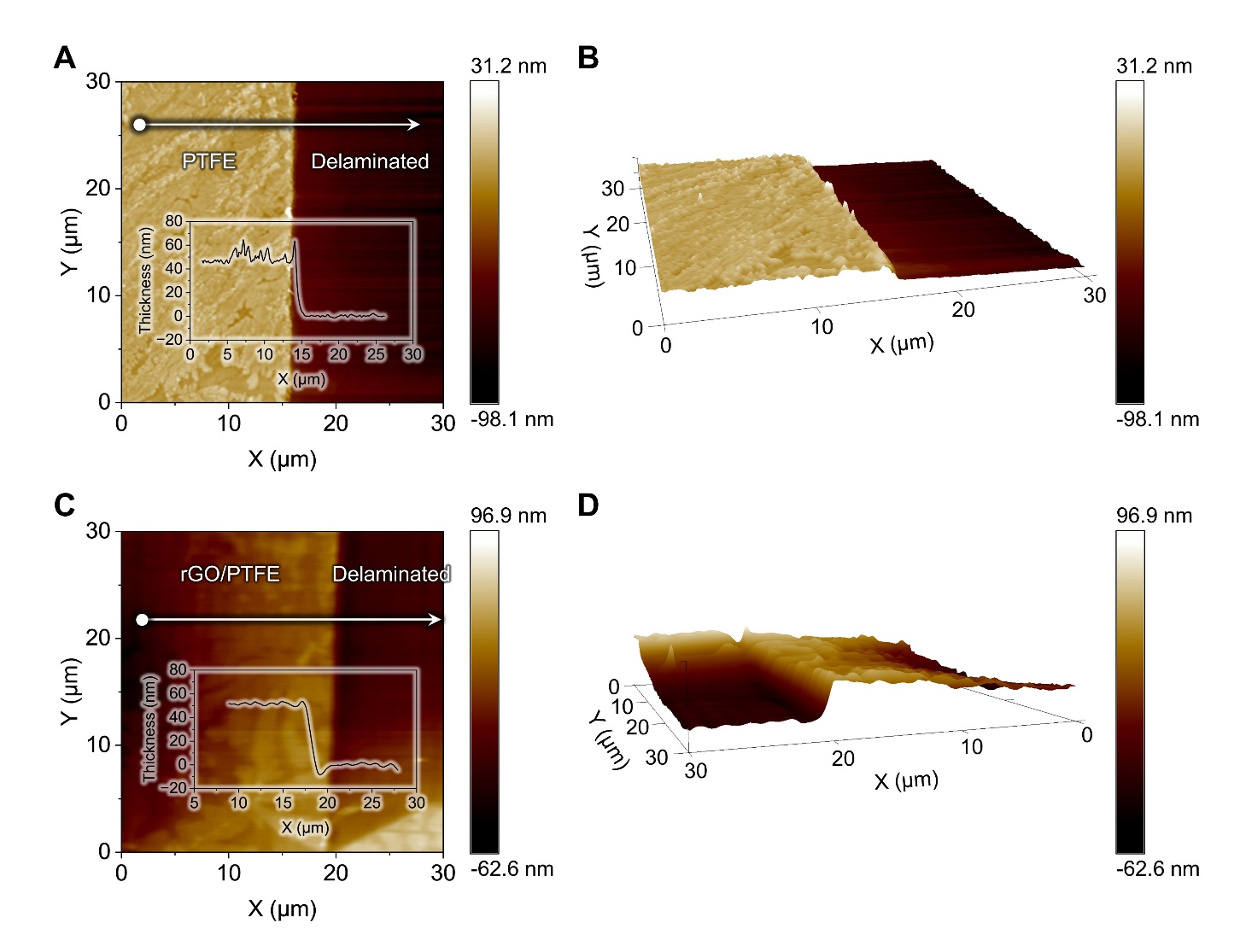
**

**Figure S15. Topographical analysis of rGO/PTFE nanocomposite films using atomic force microscopy (AFM).** (A) AFM height image of the PTFE film (EATS-P7-3.0), showing a clear boundary with the delaminated region and corresponding thickness profile. (B) 3D AFM rendering of the same PTFE film, visualizing its surface morphology. (C) AFM height image of the rGO/PTFE film (EATS-P4-7.0), with thickness profile across the delaminated interface. (D) 3D AFM rendering of the same rGO/PTFE film.

**
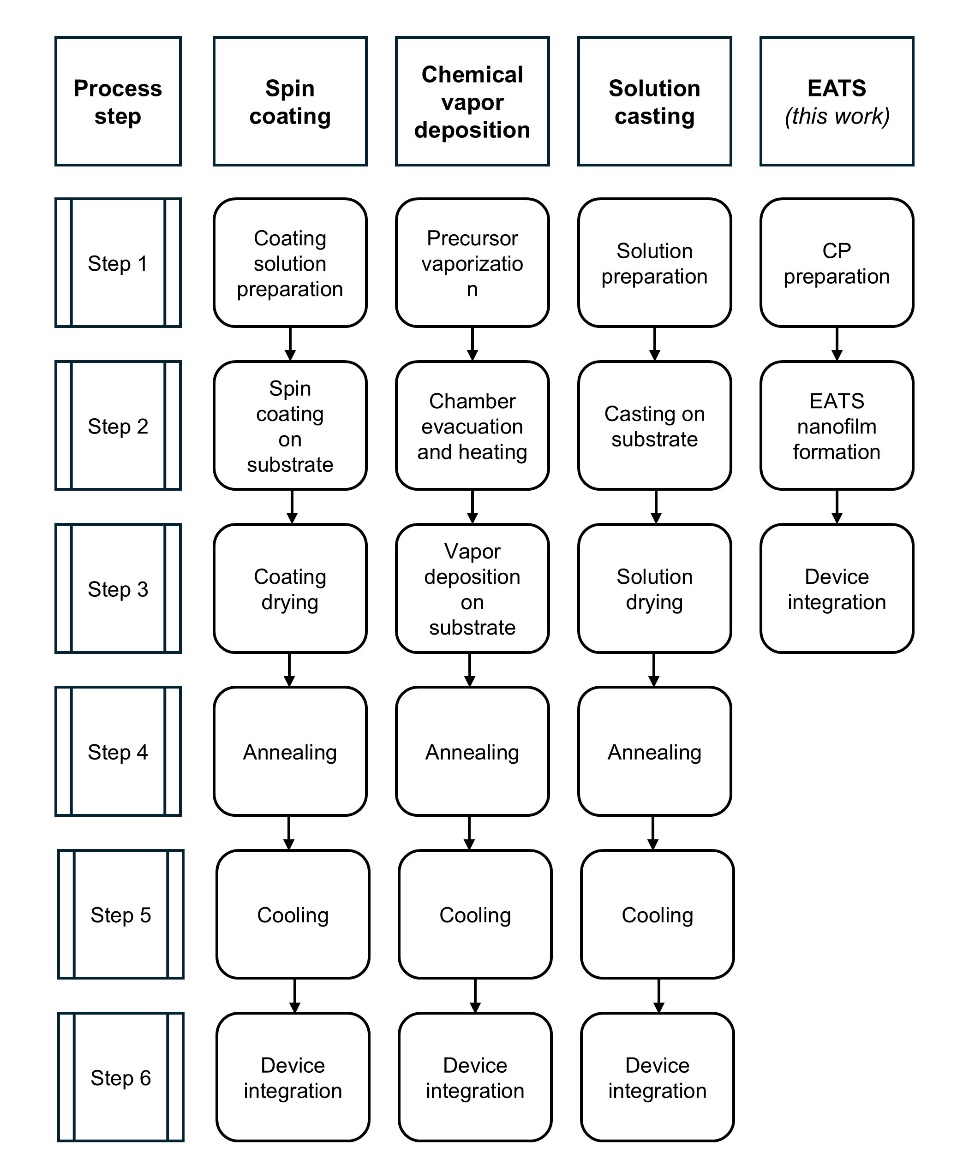
**

**Figure S16. Comparison of the fabrication workflow for conventional thin-film deposition techniques versus the EATS process.**

**Table S1. Comparison of key processing parameters, material compatibility, and scalability between conventional fabrication methods and the EATS process.**

| **Category** | **Spin coating** | **Chemical vapor deposition** | **Solution casting** | **EATS** *(this work)* |
| --- | --- | --- | --- | --- |
| **Process complexity** | Sequential coating–drying cycles and layer stacking | Vapor-phase process, precise control required | Multiple casting-drying steps | Single-step process |
| **Thermal conditions** | Low–moderate temperature  (global heating) | High temperature (global heating) | Low–moderate temperature  (global heating) | Localized, transient high-temperature heating |
| **Main processing time** | Several hours (multi-step) | Several hours (multi-step) | Several hours (multi-step) | <10 s  (single step) |
| **Atmosphere** | Ambient air | Vacuum or  controlled gas | Ambient air | Ambient air |
| **Input energy** | Low–medium power (hotplate or oven) | Long-term high thermal input (furnace or heater) | Low–medium power (extended evaporation) | Short electrical pulse  (Joule heating) |
| **Chemical  conditions** | Organic solvents, polymeric binders | Gaseous precursors, hazardous or corrosive | Solvent-based | No chemical reagents required |
| **Film uniformity  and scalability** | Limited by surface tension and solvent evaporation | Excellent local control, poor large-area uniformity | Thickness gradients during drying | Uniform thin-film formation with large-area scalability |
| **Post-processing** | Drying, annealing, cooling | Precise cooling, annealing | Drying, annealing, cooling | No post-processing required |

**
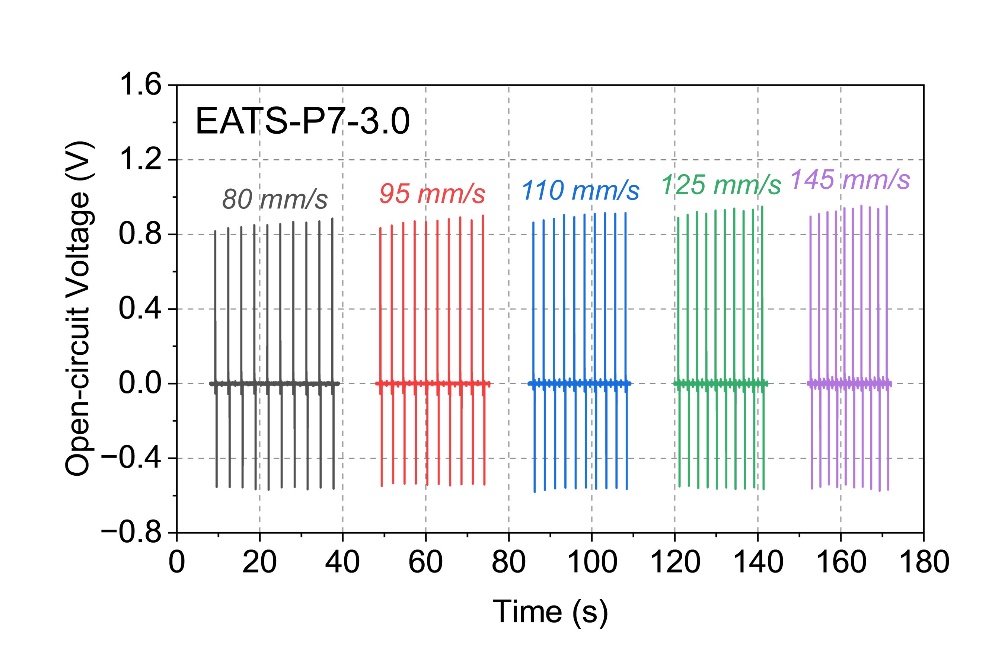
**

**Figure S17. Open-circuit voltage (OCV) profiles of a triboelectric nanogenerator (TENG) device fabricated under EATS-P7-3.0, measured at stimulus velocities of 80, 95, 110, 125, and 145 mm/s, demonstrating velocity-dependent electrical response characteristics.**

**
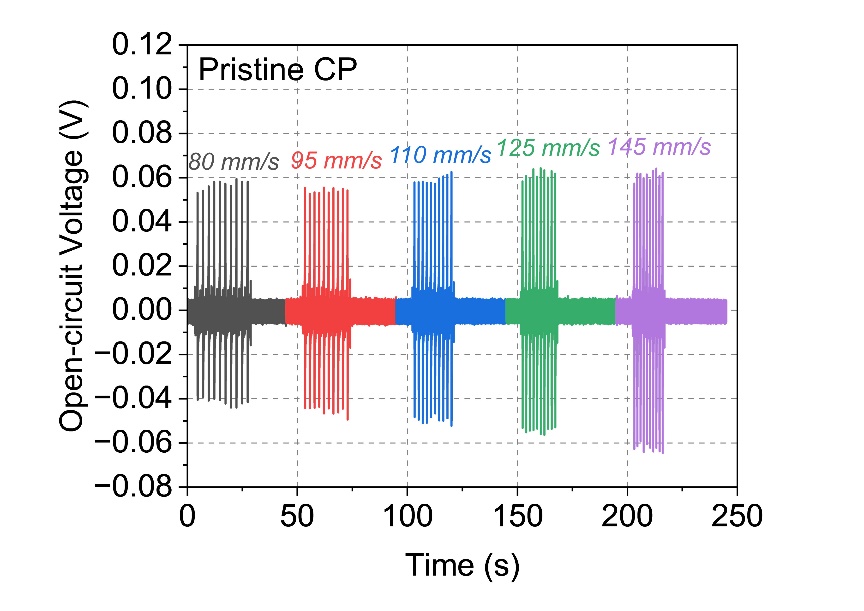
**

**Figure S18. OCV profiles of a TENG device using pristine CP, measured at stimulus velocities of 80, 95, 110, 125, and 145 mm/s.** The device exhibits only small and fluctuating outputs with weak velocity dependence, highlighting the limited triboelectric performance of pristine CP compared with the EATS-derived films.

**
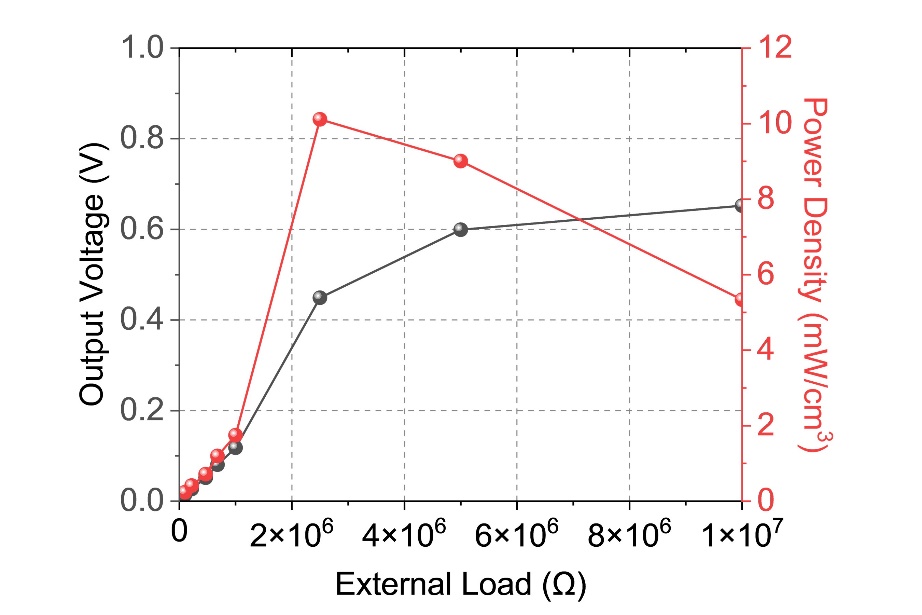
**

**Figure S19. External load matching characteristics of the EATS-P7-3.0 device.** Output voltage and power density were measured at 145 mm/s across varying external load resistances ranging from 100 kΩ to 10 MΩ, demonstrating optimal power generation behavior.

**Table S2. Comparison of power generation performance of the EATS-fabricated TENG device with previously reported triboelectric nanogenerators using PTFE-based materials.**

| Materials | Fabrication method | Dimension | Power density | Durability | Ref. |
| --- | --- | --- | --- | --- | --- |
| PTFE/graphene nanofiber yarn | Electrospinning, sintering, weaving | 2×2 cm^2^ | 0.104 mW/cm^2^ | 10,000 cycles | ^[1]^ |
| PTFE/Nylon | Expansion machine | 1 cm^2^ | 1.01 mW/cm^2^ | 10,000 cycles | ^[2]^ |
| Graphene nanosheet /PTFE film | Micro-injection molding | 3×3 cm^2^ | 0.39 mW/cm^3^ | 17.7%  over 150,000 cycles | ^[3]^ |
| rGO/PTFE | Solvent casting | 0.75 cm^2^ | 0.085 mW/cm^3^ | 100,000 cycles | ^[4]^ |
| PTFE | Stacking | 50 cm^2^ | 0.00756 mW/cm^2^ | N/A | ^[5]^ |
| PDMS/PTFE | Spin coating | 2×2 cm^2^ | 0.3 mW/m^2^ | 10,000 cycles | ^[6]^ |
| PDMS/PTFE | 3D printing | N/A | N/A (OCV~150 V / SCC~1.5 μA) | 6,000 cycles | ^[7]^ |
| PTFE/PVDF Nanofibers | Electrospinning, solution casting | 2.5×2.5 cm^2^ | 0.176 mW/cm^2^ | 100,000 cycles | ^[8]^ |
| PTFE (EATS-P7-3.0) | One-step EATS | 1×1 cm^2^ | 10.11 mW/cm^3^ | 2.8% over 10,000 cycles | *This work* |

Abbreviations: PTFE, Polytetrafluoroethylene; rGO, Reduced graphene oxide; PDMS, Polydimethylsiloxane; PVDF, Polyvinylidene fluoride

**
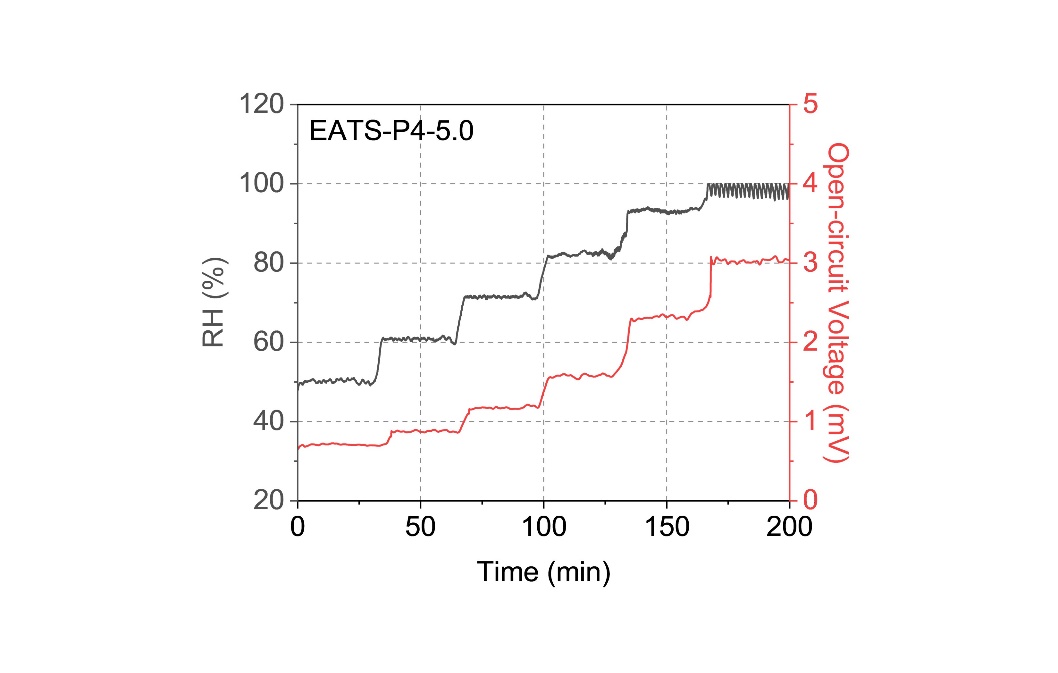
**

**Figure S20. Raw profiles of open-circuit voltage (OCV) and relative humidity (RH) for the calibration of the EATS-P4-5.0-based humidity sensor.** RH was gradually increased from 30% to 100%, and the corresponding OCV exhibited a clear stepwise increase in response to rising humidity levels.


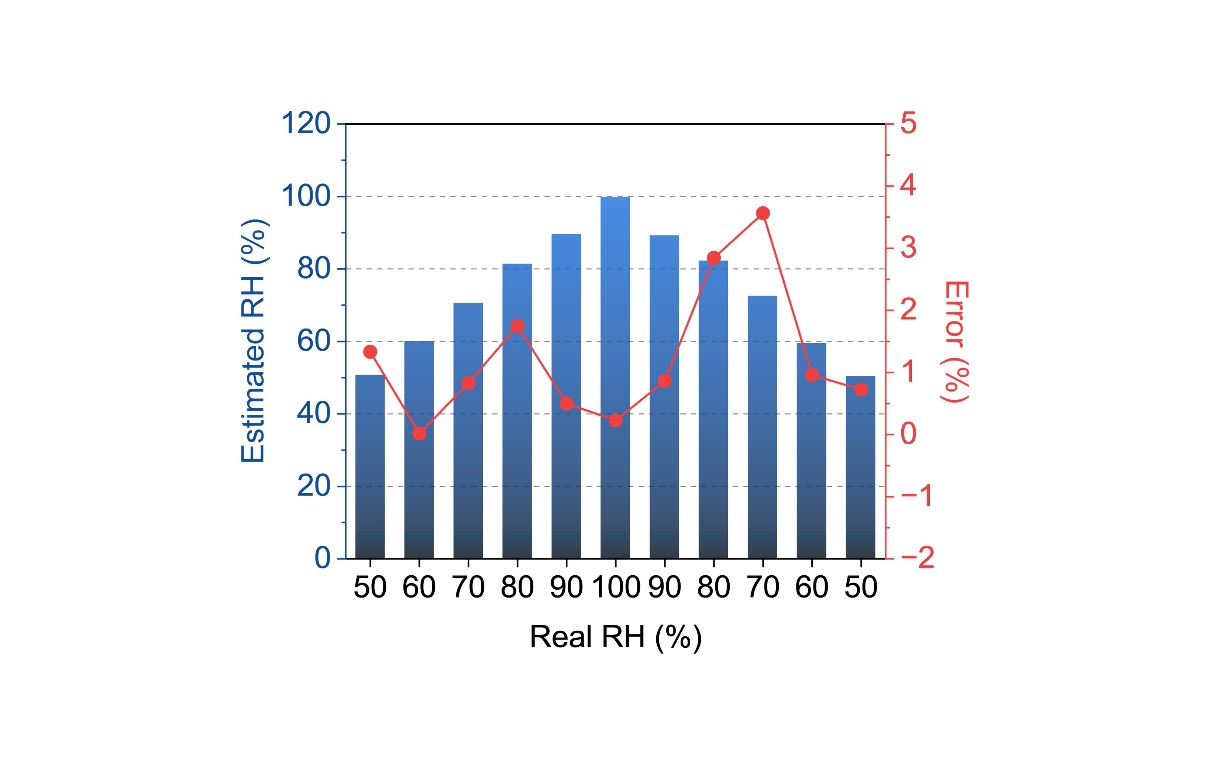


**Figure S21. Estimated RH values obtained from the EATS-P4-5.0 humidity sensor and the corresponding percentage errors, measured as the RH was sequentially varied from 50% to 100% and back to 50%.**


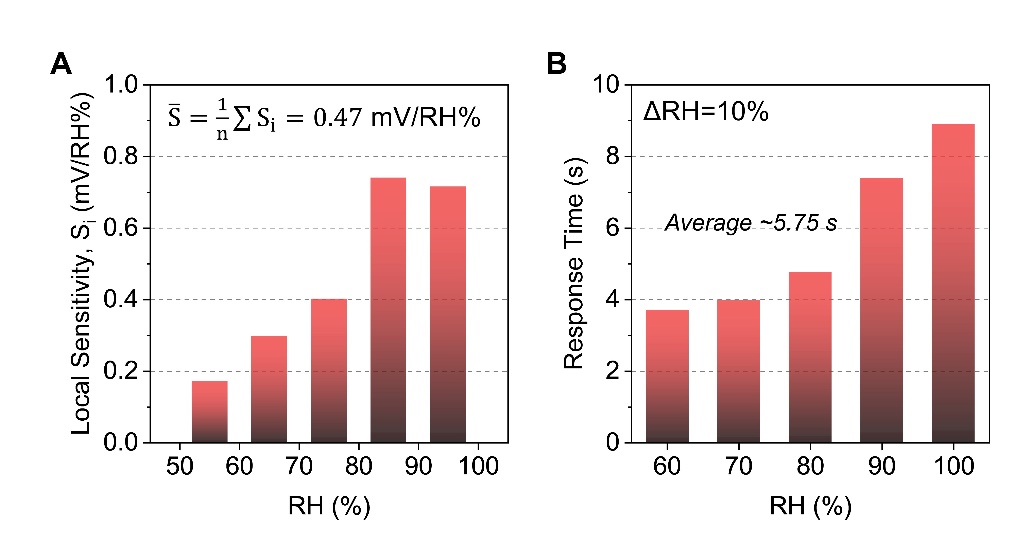


**Figure S22. Sensitivity and response time of the EATS-P4-5.0 humidity sensor.** (A) Local sensitivity (S_i_) extracted from consecutive RH intervals (50–100% RH), with an average value of 0.47 mV/RH%. (B) Response time for each 10% RH step (ΔRH = 10%), determined as the duration required to reach 99.3% of the steady-state signal (5τ), showing an average of 5.75 s.

**Note S3. Calculation of humidity sensing sensitivity.**

The local sensitivity (S_i_) of the EATS-based humidity sensor was determined from the voltage response at consecutive RH levels as follows:

$S_{i}=\frac{V_{i+1}-V_{i}}{\mathrm{RH}_{i+1}-\mathrm{RH}_{i}}$ (S1)

where V_i_ and V_i+1_ are the steady-state output voltages measured at two adjacent RH levels, RH_i_ and RH_i+1_, respectively.

The average sensitivity ($\bar{S}$) was calculated as the arithmetic mean of all local sensitivities:

$\bar{S}=\frac{1}{n}\sum_{i=1}^{n} S_{i}$ (S2)

where n is the number of RH intervals considered.


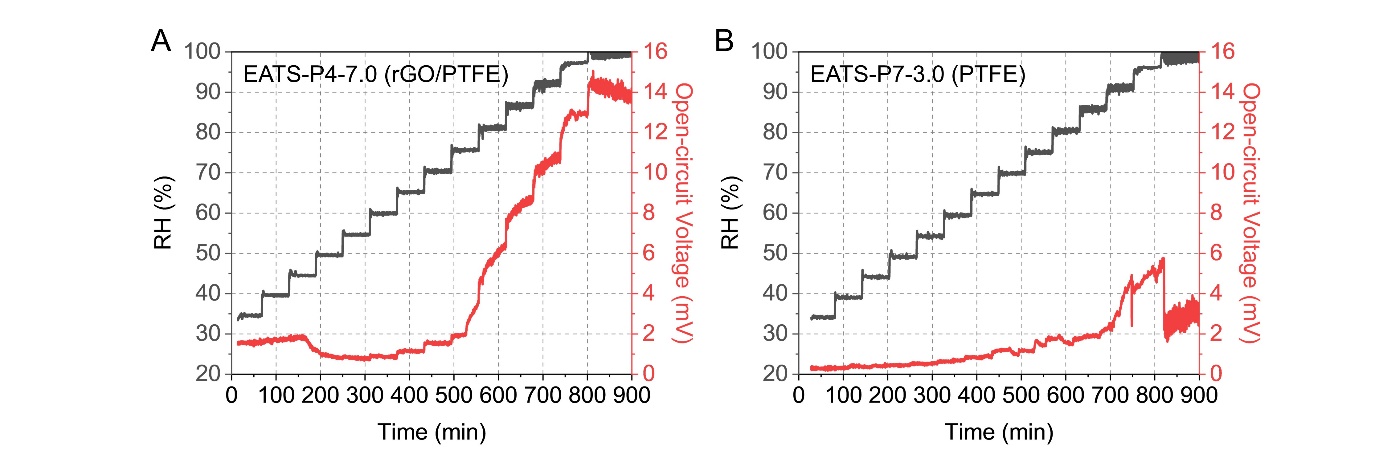


**Figure S23. OCV and RH profiles of the rGO/PTFE- and PTFE-based devices.** (A) EATS-P4-7.0 (rGO/PTFE) and (B) EATS-P7-3.0 (PTFE) devices were tested under relative humidity gradually increased from 35 % to 100 % in 5 % increments, each maintained for 60 min.

**
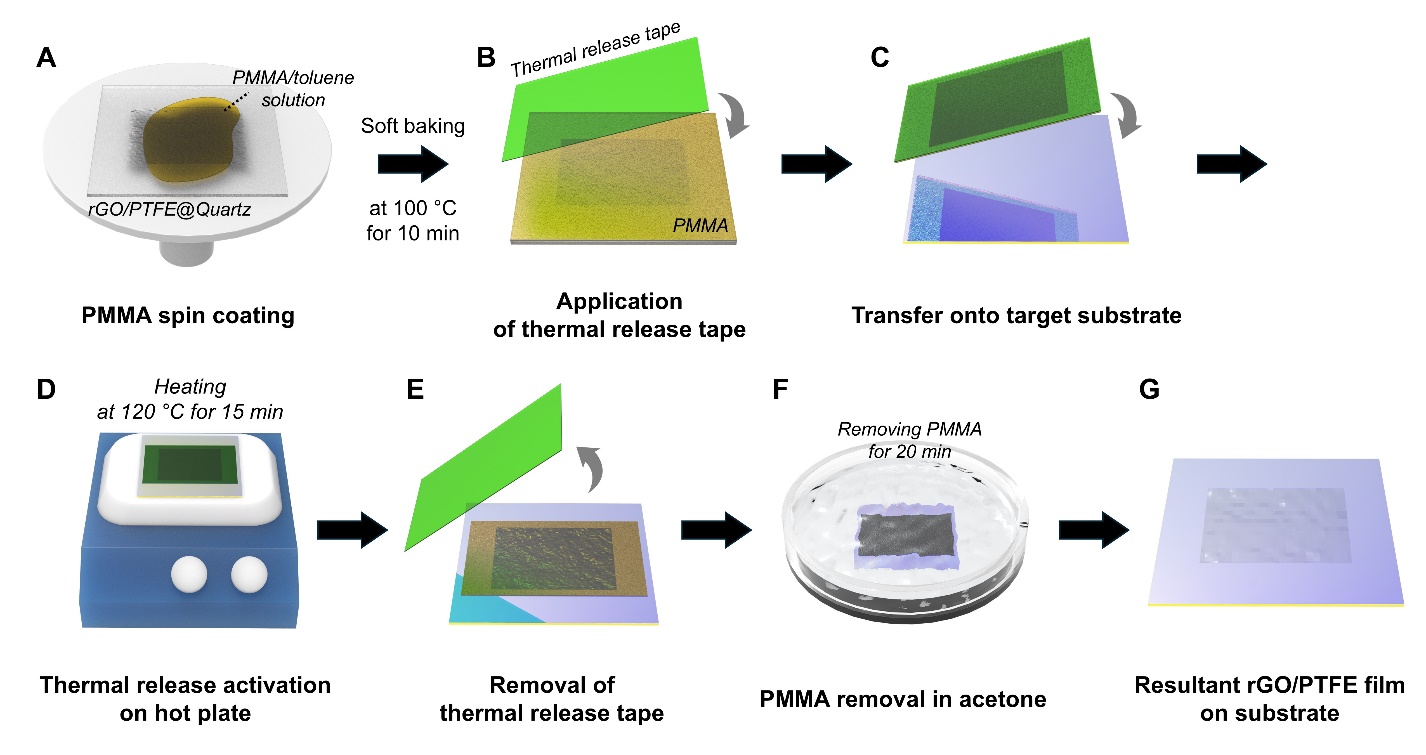
**

**Figure S24. Schematic illustration of the process for direct transfer of rGO/PTFE films onto target substrates.** (A) Spin coating of a 10 wt% polymethyl methacrylate (PMMA) solution in toluene onto rGO/PTFE films on a quartz substrate, performed at 500 rpm for 10 s followed by 5000 rpm for 45 s. (B) Soft baking at 100 °C for 10 min in an oven, followed by application of thermal release tape onto the PMMA/rGO/PTFE stack. (C) Transfer of the film onto a target substrate using the attached thermal release tape. (D) Thermal activation of the release tape by heating at 120 °C for 15 min on hot plate. (E) Removal of the thermal release tape, leaving the PMMA/rGO/PTFE film on the substrate. (F) Removal of the PMMA layer by soaking in acetone for 20 min. (G) Resultant rGO/PTFE film conformally transferred onto the target substrate.

**
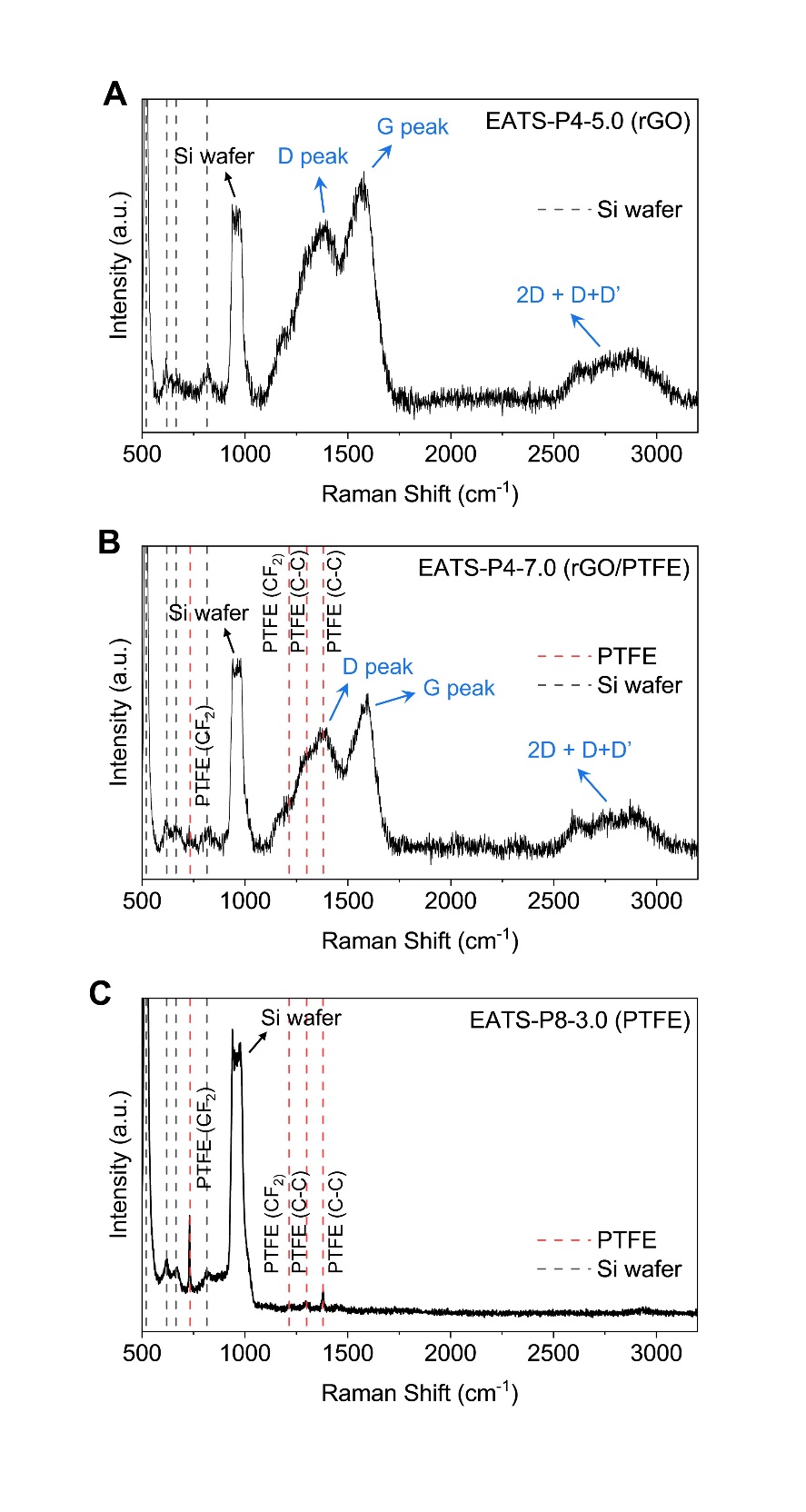
**

**Figure S25. Raman spectra of rGO, rGO/PTFE, and PTFE films transferred onto silicon wafers.** Spectra correspond to films fabricated under (A) EATS-P4-5.0 (rGO), (B) EATS-P4-7.0 (rGO/PTFE), and (C) EATS-P8-3.0 (PTFE) conditions. Characteristic peaks of the silicon wafer, rGO (D, G, 2D, and D+D’ peaks), and PTFE are indicated by dashed lines and arrows.

**Table S3. Comparison of key operational parameters and PTFE decomposition behavior between the flash Joule heating (FJH) system and the EATS process.**

| Parameter | FJH^[9]^ | EATS (*this work*) |
| --- | --- | --- |
| PTFE loading | High (10–40 wt%) | Low (~5 wt%) |
| Structural confinement | Powder bed | PTFE with highly porous carbon paper |
| Atmosphere | Low-O_2_ / quasi-inert | High porosity (~78%) in Open air Condition |
| Joule heating time scale | 10–50 ms | 400-2000 ms |
| Total Joule heating  energy per mass | ~10–17 kJ/g | ~29–42 kJ/g |
| Decomposition outcome | Gas + solid F-carbon residue | Near-complete volatilization + oxidation of CP |

**References**

[1] S. Qiu, X. Teng, Y. Zhang, X. Wang, K. Chen, J. Zhao, Q. Huang, *Chemical Engineering Journal* **2025**, 505, 159501.

[2] Z. Zhang, Y. Xu, D. Wang, H. Yang, J. Guo, L.-S. Turng, *Nano Energy* **2019**, 60, 903.

[3] P. Yang, P. Wang, D. Diao, *ACS Applied Electronic Materials* **2022**, 4, 2839.

[4] H. J. Hwang, J. S. Yeon, Y. Jung, H. S. Park, D. Choi, *Small* **2021**, 17, 1903089.

[5] D. Zhang, Z. Xu, Z. Yang, X. Song, *Nano Energy* **2020**, 67, 104251.

[6] G.-Z. Li, G.-G. Wang, D.-M. Ye, X.-W. Zhang, Z.-Q. Lin, H.-L. Zhou, F. Li, B.-L. Wang, J.-C. Han, *Advanced Electronic Materials* **2019**, 5, 1800846.

[7] R. Zheng, Y. Chen, H. Chi, H. Qiu, H. Xue, H. Bai, *ACS Applied Materials & Interfaces* **2020**, 12, 57441.

[8] Z. Yang, X. Zhang, T. Deng, G. Xiang, *ACS Applied Materials & Interfaces* **2023**, 15, 52696.

[9] W. Chen, J. T. Li, Z. Wang, W. A. Algozeeb, D. X. Luong, C. Kittrell, E. A. McHugh, P. A. Advincula, K. M. Wyss, J. L. Beckham, M. G. Stanford, B. Jiang, J. M. Tour, *ACS Nano* **2021**, 15, 11158.
